# Spectrum of γ-Secretase dysfunction as a unifying predictor of ADAD age at onset across *PSEN1*, *PSEN2* and *APP* causal genes

**DOI:** 10.1186/s13024-025-00832-1

**Published:** 2025-04-26

**Authors:** Sara Gutiérrez Fernández, Cristina Gan Oria, Dieter Petit, Wim Annaert, John M. Ringman, Nick C. Fox, Natalie S. Ryan, Lucía Chávez-Gutiérrez

**Affiliations:** 1 VIB-KU Leuven Center for Brain & Disease Research, Herestraat 49 Box 602, Louvain, 3000 Belgium; 2 Department of Neurosciences, Leuven Brain Institute, KU Leuven, Herestraat 49 Box 602, Louvain, 3000 Belgium; 3 Department of Neurology, Alzheimer’s Disease Research Center, Center for Health Professions, University of Southern California, 1520 Alcazar Street, Suite 210, Los Angeles, CA 90033 USA; 4 Dementia Research Institute at UCL, Queen Square, London, WC1 N 3BG UK; 5 Dementia Research Centre, Department of Neurodegenerative Disease, UCL Queen Square Institute of Neurology, Queen Square, London, WC1 N 3BG UK

**Keywords:** Presenilin, APP, Amyloid-β, Age at dementia onset and Familial Alzheimer’s disease, Autosomal dominant Alzheimer’s disease

## Abstract

**Background:**

Autosomal Dominant Alzheimer's Disease (ADAD), caused by mutations in Presenilins (*PSEN1/2*) and Amyloid Precursor Protein (*APP*) genes, typically manifests with early onset (< 65 years). Age at symptom onset (AAO) is relatively consistent among carriers of the same *PSEN1* mutation, but more variable for *PSEN2* and *APP* variants, with these mutations associated with later AAOs than *PSEN1*. Understanding this clinical variability is crucial for understanding disease mechanisms, developing predictive models and tailored interventions in ADAD, with potential implications for sporadic AD.

**Methods:**

We performed biochemical assessment of γ-secretase dysfunction on 28 PSEN2 and 19 APP mutations, including disease-associated, unclear and benign variants. This analysis has been valuable in the assessment of *PSEN1* variant pathogenicity, disease onset and progression.

**Results:**

Our analysis reveals linear correlations between the molecular composition of Aβ profiles and AAO for both *PSEN2* (R^2^ = 0.52) and *APP* (R^2^ = 0.69) mutations. The integration of PSEN1, PSEN2 and APP correlation data shows parallel but shifted lines, suggesting a common pathogenic mechanism with gene-specific shifts in onset. We found overall “delays” in AAOs of 27 years for PSEN2 and 8 years for APP variants, compared to PSEN1. Notably, extremely inactivating *PSEN1* variants delayed onset, suggesting that reduced contribution to brain APP processing underlies the later onset of PSEN2 variants.

**Conclusion:**

This study supports a unified model of ADAD pathogenesis wherein γ-secretase dysfunction and the resulting shifts in Aβ profiles are central to disease onset across all causal genes. While similar shifts in Aβ occur across causal genes, their impact on AAO varies in the function of their contribution to APP processing in the brain. This biochemical analysis establishes quantitative relationships that enable predictive AAO modelling with implications for clinical practice and genetic research. Our findings also support the development of therapeutic strategies modulating γ-secretase across different genetic ADAD forms and potentially more broadly in AD.

**Supplementary Information:**

The online version contains supplementary material available at 10.1186/s13024-025-00832-1.

## Introduction

Alzheimer's disease (AD) is a progressive disorder characterized by cognitive decline, brain atrophy, and molecular pathology defined by the extracellular accumulation of misfolded amyloid-beta (Aβ) peptides, intracellular aggregation of hyperphosphorylated tau protein and neuroinflammation in the brain [1]. While most AD cases are late-onset and sporadic, a small percentage (< 1%) are caused by mutations in the Presenilin 1 (*PSEN1*), Presenilin 2 (*PSEN2*), and Amyloid Precursor Protein (*APP*) genes [2]. This autosomal dominant AD (ADAD), which is typically characterized by an early age at symptom onset (AAO) (< 65 years) [3], provides a valuable model to elucidate the underlying pathogenic mechanisms and offers opportunities for the assessment and development of early intervention and targeted therapies [4].

**Fig. 1:**
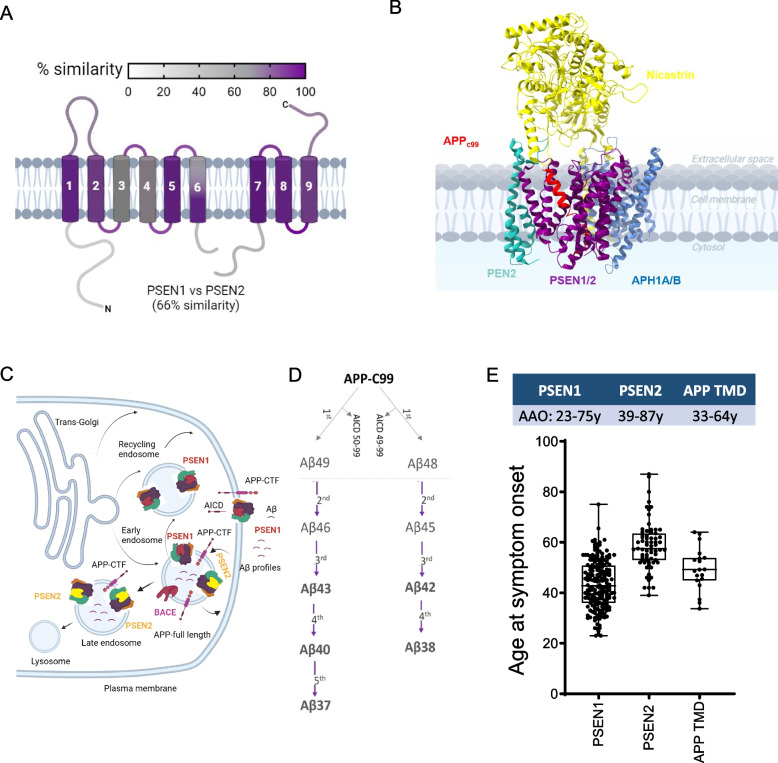
Mutations in PSEN1, PSEN2, and APP TMD cause ADAD with varying AAOs. **A** Schematic showing PSEN1 and PSEN2 isoforms, which share 66% homology. The colour gradient displays local homology between PSEN1 and PSEN2 (based on NCBI's Basic Local Alignment Search Tool (BLAST) using BLOSUM 62 matrix). **B** GSEC-APP_C99_ co-structure (PDB: 8X54). PSEN (purple) forms the catalytic subunit, while Nicastrin (yellow), PEN2 (green), and APH1 A/B (blue) are essential subunits of the GSEC complex. APP_C99_ is the direct substrate of GSEC (red). **C** Schematic representation of PSEN1- and PSEN2-type GSEC complex subcellular localizations. PSEN1-type GSEC complexes (red) are broadly distributed (both in the plasma membrane and in late endosomes), while PSEN2-type GSECs (in yellow) are restricted to late endosomes. Created in BioRender. Gutierrez Fernandez, S. (2025) https://BioRender.com/v47y310 **D**. APP cleavage by BACE1 generates APP_C99_, the direct GSEC substrate. The initial GSEC-mediated cut (endopeptidase activity) releases AICD_50-99_ or AICD_49-99_ and generates longer Aβ fragments (Aβ48 or Aβ49). These fragments undergo sequential γ-cleavages to produce Aβ peptides of various lengths. Pathogenic mutations destabilize the GSEC-APP/Aβ complex, reducing sequential cleavage efficiency (processivity) and increasing the release of longer, more toxic Aβ peptides. **E** Age at symptom onset (AAO) associated with mutations in PSEN1, PSEN2, and APP genes. PSEN1 harbours most ADAD mutations with broadly distributed AAOs (23 - 75y). PSEN2 and APP mutations are associated with later onsets (33 - 64y and 39 - 87y, respectively). Box plots show the median (centre line) and 25 - 75 percentiles. Dots represent individual mutations plotted as averaged mean ± SD. Data was sourced from Alzforum database (https://www.alzforum.org/mutations) and literature (see Supplementary Tables S1, S2 and S3)

PSEN1 and PSEN2 are highly homologous isoforms (Fig. 1A) that serve as the catalytic subunit of the γ-secretase intramembrane protease complex (GSEC) (Fig. 1B). Despite their functional similarities, they differ in their enzymatic efficiencies [5] and subcellular localization, with PSEN2 being less efficient than PSEN1 and restricted to late endosomes/lysosomes, while PSEN1 localizes to both plasma membrane and endosomal compartments (Fig. 1C) [6].

GSECs generate Aβ peptides from APP [7]. The initial cleavage of APP by the β-secretase (BACE1) releases APP ectodomain and generates a transmembrane C-terminal fragment of 99 amino acids (aa) in length (APP_C99_) [8], which is subsequently proteolyzed by GSECs. The initial (endopeptidase) GSEC-mediated cleavage releases a soluble domain (AICD) intracellularly and generates either Aβ49 or Aβ48 peptides (49 or 48 aa, respectively) that remain bound to the enzyme. The Aβ49/48 peptides undergo sequential processing along two product lines (referred to as GSEC processivity), until the secretion of a shortened Aβ_n_ peptide to the extracellular or luminal environment ends the processive GSEC proteolysis [7] (Fig. 1D). ADAD-linked mutations in PSEN1/PSEN2 (GSEC) and APP genes alter Aβ production and/or peptide properties. Most notably, ADAD-linked *PSEN1, PSEN2* and some *APP* variants lower the efficiency of the sequential GSEC processing [9, 10] by destabilizing GSEC-APP/Aβ (enzyme–substrate) interactions (Fig. 1D) [11]. As a result, these mutations cause relative increases in longer Aβ42 [12, 13, 14, 15, 16] and Aβ43 peptides [17, 18, 19], which are key drivers of amyloid seeding leading to early pathogenic cascades [17]. Of note, ADAD-linked variants in the extracellular region of APP_C99_ may increase the aggregation propensities of (mutant) Aβ peptides, while keeping the spectrum of Aβ lengths (Aβ profiles) unaltered [2, 20]. This variability in APP mutation effects further adds complexity to the ADAD pathogenicity.

While the amyloid hypothesis has faced challenges, including failed Aβ-targeting clinical trials [21], recent anti-amyloid immunotherapy trials have shown promise in slowing disease progression, leading to regulatory approvals [22]. These successes, albeit still limited, not only validate the therapeutic potential of targeting Aβ but also emphasize the need for a deeper molecular understanding of the early phases of AD pathogenesis to enable the development of more effective therapies and to identify optimal treatment windows.

Our research on *PSEN1* mutations [23] has demonstrated a strong linear correlation between the composition of Aβ profiles generated in vitro by mutant GSECs (PSEN1) and patient AAO. Specifically, we found that mutation-driven changes in the GSEC processivity, quantified by the short-to-long Aβ(37 + 38 + 40)/(42 + 43) peptide ratio, relative to the wild type (WT), strongly correlate with AAO (R^2^ = 0.78). More recently, Schultz et al. [24] extended these observations to 161 *PSEN1* variants, revealing linear correlations between GSEC processivity, AAO, and multiple clinical and core biomarker data (grey matter volume, amyloid PET, Aβ42/40 ratio, phosphorylated tau in CSF). These findings emphasize the pathogenicity of imbalances in Aβ peptide ratios, rather than simple increases in specific peptides – a notion that prompts a re-evaluation of prevalent concepts in the field.

The relationships between GSEC processivity and disease onset for mutations in *PSEN2* and *APP* remain less clear. Clinical presentation and AAO vary depending on the affected gene [3, 25], with *PSEN2* and *APP* mutation carriers typically presenting significantly later onsets than those with *PSEN1* mutations (Fig. 1E). AAO is relatively consistent among carriers of the same *PSEN1* variant (Table 1 in Petit et al., 2022 [23]), while *PSEN2* mutation carriers can exhibit remarkably wide variations in AAO, even within families carrying the same mutation (Table 1). Intriguingly, PSEN2-type GSECs generate Aβ profiles that are enriched in longer Aβ peptides, compared to PSEN1-type [5, 6, 19]; yet, carriers of *PSEN2* variants develop dementia at later ages. Similar to *PSEN2* mutations, *APP* variants are associated with relatively variable AAOs [3, 25] (Table 2) (Fig. 1E). The clinical heterogeneity in *PSEN2* and *APP* mutation carriers poses significant challenges for genetic counselling and prognostic predictions.

**Table 1:** Analysed mutations in *PSEN2*, their location and associated AAOs

	**PSEN2 mutation**	**Position in PSEN2**	**Mean AAO (range)**	**# Cases**	**Classification**
1	A85V	N-term	61.5 (55.0–71.0)	4	Not classified
**2**	**T122P**	**Loop 1**	**47.3 (46.0–50.0)**	**3**	**Likely pathogenic**
3	P123L	Loop 1	57.0	1	Not classified
4	E126K	Loop 1	53.5 (48.0–59.0)	2	Not classified
5	S130L	Loop 1	65.2 (51.0–81.0)	9	Uncertain significance
6	N141D	TMD 2	59.0	1	Not classified
**7**	**N141I**	**TMD 2**	**56.6 (40.0–76.0)**	87/101 (*)	**Pathogenic**
8	N141S	TMD 2	52.0	1	Not classified
**9**	**N141Y**	**TMD 2**	**46.0 (43.0–49.0)**	**2**	**Likely pathogenic**
10	I149T	TMD 2	63.0	1	Not classified
11	K161R	Loop 2	65.0	1	Not classified
12	H169N	Loop 2	62.5 (56.0–68.0)	4	Uncertain significance
13	S175C	TMD 3	62.0 (60.0–65.0)	3	Not classified
14	S175F	TMD 3	53.0 (49.0–58.0)	3	Not classified
15	G212V	TMD 4	61.5 (60.0–65.0)	4	Not classified
16	I235F	TMD5	57.0	1	Not classified
17	L238F	TMD 5	60.0 (49.0–74.0)	4	Uncertain significance
**18**	**M239I**	**TMD 5**	**50.1 (30.0–58.0)**	16/20 (*)	**Pathogenic**
**19**	**M239V**	**TMD 5**	**57.2 (45.0–83.0)**	28/44 (*)	**Pathogenic**
**20**	**M239T**	**TMD 5**	**52.0 (47.0–59.0)**	**3**	**Pathogenic**
**21**	**R284G**	**Loop 6**	**57.5 (57.0–58.0)**	**2**	**Likely pathogenic**
**22**	**M298T**	**Loop 6**	**57.2 (56.0–59.0)**	**5**	**Likely pathogenic**
23	A379D	Loop 7	55.0	1	Not classified
24	P69A	N-term	74.0	1	Benign
25	R71W	N-term	63.4 (55.0–75.0)	9/18 (*)	Benign
26	V214L	TMD 4	57.2 (42.0–69.0)	11/13 (*)	Benign
27	P334A	Loop 6	Not reported	1	Benign
28	T421M	TMD 9	55.0	1	Benign

This table summarizes analysed PSEN2 mutations, including their positions in the PSEN2 primary structure, associated AAOs, number of cases, and classification. AAOs were obtained from the Alzforum database and literature (see Supplementary Table S1). Mutations reported as pathogenic/likely pathogenic are highlighted in bold. P69A, R71W, V214L, P334A, and T421M substitutions, reported as benign, were selected as controls. Abbreviations: Transmembrane domain (TMD), extracellular loop between TMD1 and TMD2 in PSEN (Loop 1), N-terminal region (N-term). (*) Number of carriers included in this study vs total reported cases according to the Alzforum database

**Table 2:** Analysed mutations in *APP* TMD and associated AAOs

	**APP TMD mutation**	**Mean AAO (range)**	**# Cases**	**Classification**
1	L705V	63.4 (50.0–72.0)	5	CAA: Pathogenic
2	A713T	61.3 (49.0–76.0)	13/16 (*)	Uncertain significance
**3**	**T714A**	**53.0 (44.0–69.0)**	10/11 (*)	**Pathogenic**
**4**	**T714I**	**37.4 (32.0–42.0)**	**7**	**Pathogenic**
**5**	**V715A**	**48.7 (42.0–55.0)**	**6**	**Pathogenic**
**6**	**V715M**	**49.3 (41.0–60.0)**	**4**	**Pathogenic**
**7**	**I716F**	**33.7 (30.0–47.0)**	**6**	**Pathogenic**
8	I716M	64.0	1	Not classified
9	I716T	36.0	1	Not classified
10	I716V	55.7 (53.0–58.0)	3	Not classified
**11**	**V717F**	**44.9 (37.0–52.0)**	**21**	**Pathogenic**
**12**	**V717G**	**52.5 (40.0–67.0)**	**17**	**Pathogenic**
**13**	**V717I**	**53.9 (41.0–62.0)**	**61**	**Pathogenic**
**14**	**V717L**	**46.9 (35.0–59.0)**	**26**	**Pathogenic**
**15**	**T719N**	**45.5 (45.0–46.0)**	**2**	**Pathogenic**
16	T719P	43.0	1	Not classified
**17**	**M722K**	**49.2 (38.0–56.0)**	**5**	**Pathogenic**
**18**	**L723P**	**47.0 (45.0–57.0)**	**3**	**Pathogenic**
19	K724N	53.5 (52.0–55.0)	2	Not classified

This table presents APP Transmembrane Domain (APP TMD) mutations selected for analysis, their associated AAOs, number of cases, and classification. Mutation AAOs were defined according to the Alzforum database and available literature (see Supplementary Table S2). Mutations reported as pathogenic/likely pathogenic are highlighted in bold. (*) Number of carriers included in this study vs total reported cases according to the Alzforum database

The substantial clinical variability among *PSEN2* and *APP* mutation carriers underscores the need for biochemical analyses that can provide insights into variant pathogenicity and potentially predict onset independent of confounding factors. Building on our previous *PSEN1* analyses [23], we investigated whether similar relationships exist between Aβ profiles and AAO in carriers of *PSEN2* variants and *APP* transmembrane domain (TMD) mutations. As with *PSEN1*, we found that PSEN2 and APP (TMD) mutation-induced shifts in Aβ ratios correlate linearly with AAO. The integration of these results with *PSEN1* data [23] stablishes a robust quantitative framework for assessing mutation pathogenicity and predicting AAO across the three ADAD causal genes. These findings have significant implications for AAO modelling/prediction and genetic counselling, and may facilitate research aimed at identifying genetic and environmental modulators of disease onset. Moreover, our study reinforces the rationale for developing GSEC-targeted therapies with potential applications in both familial and sporadic AD.

## Results

### Characterization of PSEN2 mutations and their impact on GSEC activity

**Fig. 2:**
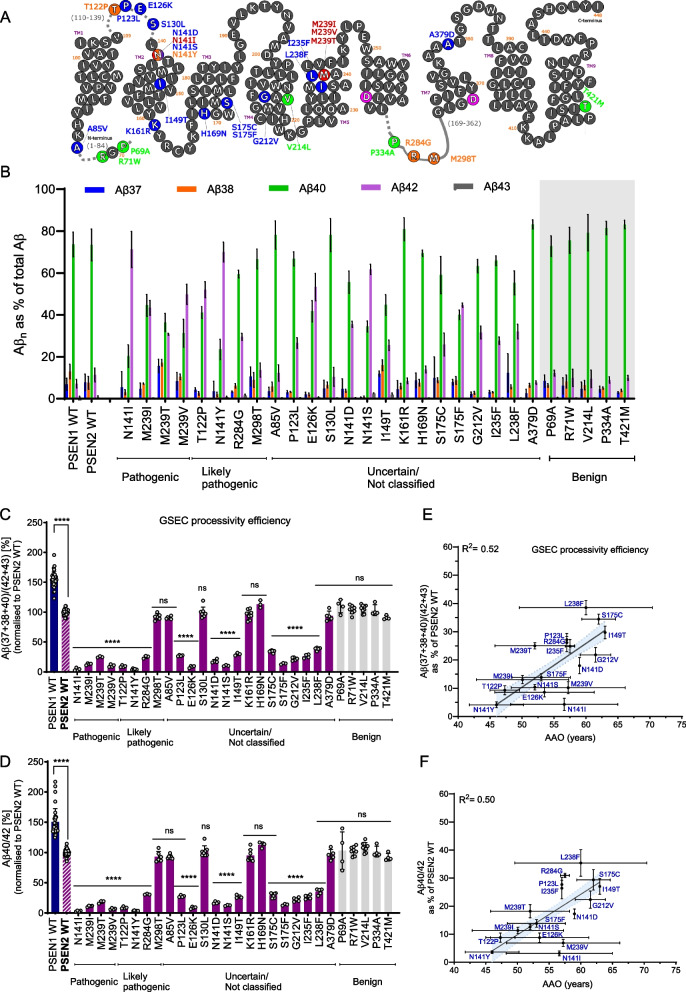
PSEN2 mutations significantly alter GSEC processivity, mirroring PSEN1 pathogenic mechanisms. **A** Schematic representation of PSEN2 primary structure highlighting residues affected by selected mutations studied in this report. Color-coding of mutations: blue (not classified/unclear significance), green (benign), red (pathogenic), orange (likely pathogenic). Mutations selected for this study are shown. Pathogenicity information taken from the Alzforum database. **B** Aβ profiles (showing the relative abundance of Aβ37, Aβ38, Aβ40, Aβ42, and Aβ43 peptides relative to total Aβ) generated by PSEN1 WT-, PSEN2 WT-, or mutant PSEN2-containing GSECs. Benign mutations (controls) are displayed on grey background. Mutations are classified based on Alzforum database in: pathogenic, likely pathogenic, uncertain significance/not classified or benign. Data presented as mean ± SD, N ≥ 3 independent experiments. **C** Efficiency of 4 th enzymatic GSEC turnover of APP_C99_ (estimate of GSEC processivity) quantified by the Aβ(37 + 38 + 40)/(42 + 43) ratio; data are normalised to PSEN2 WT (in bold and striped filled pattern). PSEN1 WT (in blue), PSEN2 WT and analysed variants in purple, benign variants (control) in light grey. Data presented as mean ± SD, N ≥ 3 independent experiments. Statistical significance determined by one-way ANOVA and Dunnett's post-hoc test compared to PSEN2 WT (p < 0.05); ****p < 0.0001, (F(DFn, DFd): F (29, 173) = 305.1). **D** Aβ40/42 ratio data normalised to PSEN2 WT (in bold and striped filled pattern). PSEN1 WT (in blue), PSEN2 WT and analysed variants in purple, benign variants (control) in light grey. Data presented as mean ± SD, N ≥ 3 independent experiments. Statistical significance determined by one-way ANOVA followed by Dunnett's post-hoc test compared to PSEN2 WT (p < 0.05); ****p < 0.0001, (F(DFn, DFd): F (29,173) = 123.1). **E** Correlation analysis between GSEC processivity (normalised to PSEN2 WT) and AAO. This analysis includes all PSEN2 variants showing significant differences compared to PSEN2 WT in the Aβ(37 + 38 + 40)/(42 + 43) ratio (panel C). Significant correlation found (equation: Y = 1.5*X – 67, R^2^ = 0.52). 95% confidence interval shown as light blue area. Error bars represent SD for the processivity ratio (x-axis) and AAO (y-axis). **F** Correlation analysis between the Aβ40/42 ratio (normalised to PSEN2 WT) and AAO. This analysis includes all PSEN2 variants showing significant differences compared to PSEN2 WT in the Aβ40/42 ratio (panel D). Significant correlation found (equation: Y = 1.4*X – 62, R^2^ = 0.50). 95% confidence interval shown as light blue area. Error bars represent SD for Aβ ratio (x-axis) and AAO (y-axis)

To gain insights into the mechanisms by which *PSEN2* mutations contribute to ADAD pathogenesis, we conducted an analysis of a total of 28 *PSEN2* mutations (Table 1), including 4 classified as ‘pathogenic’, 4 as ‘likely pathogenic’, 15 as 'not classified' or with 'unclear significance' and 5 ‘benign’ variants. These variants span across the PSEN2 structure and are depicted in red, orange, blue and green in Fig. 2A, respectively. To evaluate PSEN2 function, we established WT and mutant PSEN2 cell lines by rescuing the expression of the respective human PSEN2 in *psen1/psen2* deficient mouse embryonic fibroblasts, as described in Petit et al*.* 2022 [23]. We note that eight of these PSEN2 variants have ‘sister’ mutations in PSEN1 (same mutation in the same position), and their effects on APP processing, relative to their sister PSEN1 variants, have been previously analysed [23]. PSEN2 mutants efficiently reconstituted mature GSEC complexes (Supplementary Figure S1).To examine their effects on Aβ production, we transiently expressed human APP_C99_, the direct substrate of GSEC from which Aβ peptides are generated. PSEN2 contains an N-terminal motif that restricts its localization to the late endosomes and lysosomes [6] (Fig. 1C), resulting in the intracellular processing of APP. We therefore measured both intracellular and secreted Aβ peptide pools (sum of the Aβ37, Aβ38, Aβ40 and Aβ42) generated by cell lines expressing the pathogenic and likely-pathogenic PSEN2 variants (Supplementary Figure S2 A). We found that secreted Aβ peptides represent the largest pool generated by the tested mutant PSEN2-type GSECs, providing the most information about mutation-driven effects on Aβ profile analysis.

### PSEN2 mutation-induced shifts in GSEC processivity linearly correlate with AAO

To compare the inherent properties of PSEN1 and PSEN2-containing GSEC complexes, we first analysed the processivity of WT PSEN1 versus PSEN2 enzymes. Aβ profile analysis (Fig. 2B) showed substantial relative increases in the production of Aβ42 but, in contrast to PSEN1, most PSEN2 variants did not significantly increase Aβ43 levels. The levels of this peptide, relative to the total Aβ40 product (Aβ40 + Aβ37), were significantly increased only for the N141Y, E126K, N141S and N141D variants (Supplementary Figure S2C). To estimate GSEC processivity, we calculated the long-to-short Aβ(37 + 38 + 40)/(42 + 43) peptide ratio (Fig. 2C). Consistent with previous reports [5, 6], the WT PSEN2 cell line showed significantly lower processivity than the WT PSEN1 line. Additionally, 17 out of 28 PSEN2 variants displayed significantly lowered processivity ratios compared to WT PSEN2. Given the strong linear correlation between the Aβ40/42 ratio and AAO observed in pathogenic *PSEN1* variants [23], we also analysed this ratio (Fig. 2D). Both the processivity and Aβ40/42 ratios showed significantly lower values for all the confirmed pathogenic (N141I, M239I, M239V and M239T) and all the ‘likely pathogenic’ *PSEN2* variants, except for the M298T mutation. Furthermore, the P123L, E126K, N141D, N141S, I149T, S175C, S175F, G212V, I235F and L238F mutations, currently labelled as'not classified'or with'unclear significance'lowered both Aβ ratios. In contrast, the PSEN2- P69 A, R71W, V214L, P334A and T421M variants showed no significant changes, supporting their benign classification. Moreover, the 'uncertain' and 'not classified' *PSEN2*- A85V, S130L, K161R, H169N and A379D mutations did not show differences, relative to WT *PSEN2*, suggesting non-pathogenic roles for these variants. Interestingly, the M298T mutation, classified as ‘likely pathogenic’ by the Alzforum, did not show significant differences in both Aβ ratios, compared to *PSEN2* WT. This variant has been reported in one affected person, whose age of onset was 56 and thought not to be familial [26], one person diagnosed with mild cognitive impairment and two with AD from a family with 7 individuals affected by dementia in 2 generations in whom their genetic status was not documented [27] and one person diagnosed with dementia at age of 56y and with a positive family history of dementia [28]. Our in vitro analysis, showing no alterations in the processivity and Aβ40/42 ratios, does not support pathogenicity for the M298T variant. We note that ClinVar, another important database for genetic variants, currently describes the *PSEN2*-M298T variant as being of "unknown significance" [29].

We next assessed the correlation between the processivity Aβ(37 + 38 + 40)/(42 + 43) ratio or the Aβ40/42 ratio (both as % WT) and AAO for the 17 *PSEN2* mutations that significantly lowered these ratios. We note that the Aβ pools secreted by these 17 ‘pathogenic’ PSEN2 variants, and the M298 T mutation, were consistently found as the major ones (Supplementary Figure S2A and S2B). AAOs were extracted from the literature (Table 1 and Supplementary Table S1). We found linear correlations for both: Y = 1.5 x – 67; R^2^ = 0.52, p < 0.0001 and Y = 1.4 x – 62; R^2^ = 0.50, p < 0.0001, respectively (Figs. 2E and 2 F, respectively). These consistent results indicate that the simpler Aβ40/42 ratio provides sufficient information for the evaluation of pathogenicity and AAO in both PSEN1 and PSEN2 variants.

We also analysed the mutation effects on the GSEC product line preference by calculating the Aβ(37 + 40 + 43)/(38 + 42) ratio, which weights the products of the two different production lines. We found significant changes in this ratio for the same mutations that showed altered Aβ processing, and a weaker but significant correlation with AAO (R^2^ = 0.43, p < 0.0001) (Supplementary Figure S3A-B). In addition, we estimated the Aβ37/42 ratio, previously reported to outperform the Aβ42/40 ratio [30]. We found significant changes in the 17 *PSEN2* mutations flagged by the previous Aβ ratios, and also for the *PSEN2*- A85V and T421M variants. The analysis of PSEN1-A79V vs PSEN2-A85V sister mutations [23] showed a mild but significant processivity impairment for both sister variants relative to WT PSEN1. The Aβ37/42 ratio, though informative, should be interpreted with caution given the benign nature of the T421M variant. The analysis of the Aβ37/42 ratio – AAO relationship (T421M included) revealed a weaker though significant correlation (R^2^ = 0.21, *p* < 0.0001) (Supplementary Figure S3C-D). We also analysed the efficiency of the Aβ42 → Aβ38 cleavage by calculating the Aβ38/42 ratio. We found significant changes in 13 out of 28 mutations (Supplementary Figure S3E), with the pathogenic M239I and M239V mutations not showing significant changes compared to WT. The Aβ38/42 ratio-AAO analysis (R^2^ = 0.21, p < 0.0001) is shown in Supplementary Figure S3F. These data imply that the PSEN2 variants act by promoting the Aβ42 - 38 product line (which reduces Aβ40 production) while lowering the efficiency of the Aβ42 → Aβ38 cleavage.

### Biochemical prediction of AAOs for PSEN2 variants and comparison with clinical AAOs

**Fig. 3:**
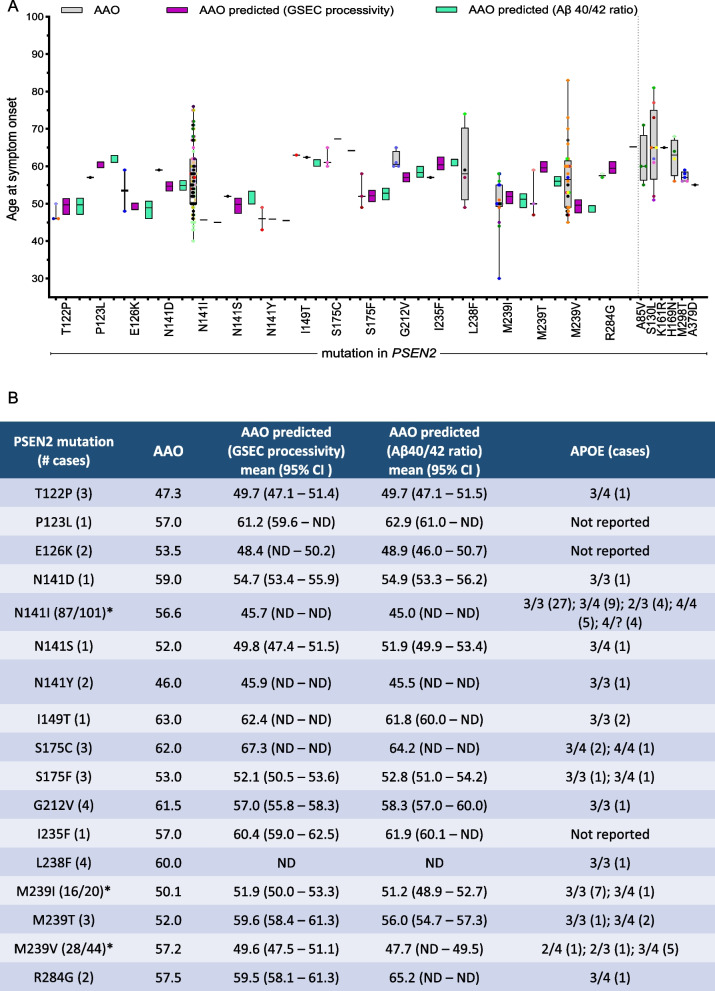
GSEC processivity and Aβ40/42 ratio predict AAO in ADAD-linked PSEN2 variants. **A** Comparison of clinical and predicted AAOs for PSEN2 mutations. Clinical AAOs for each PSEN2 mutation are shown in grey boxes (mean ± SD) with individual mutation carriers represented by coloured dots (each colour denotes one family). Purple and green boxes show predicted AAOs based on correlative data for Aβ(37 + 38 + 40)/(42 + 43) processivity ratio and AAO, or Aβ40/42 ratio and AAO, respectively (mean ± 95% CI) from correlations in Fig. 2E and F. Clinical AAOs for mutations showing no significant differences compared to PSEN2 WT in Figs. 2C/2D are shown on the right. **B** Summary table of PSEN2 data for variants that significantly altered Aβ ratios, including: mutation, number of cases, clinical AAO, predicted AAOs (based on processivity and Aβ40/42 ratios), 95% CI of predicted AAO, and APOE genotypes for reported cases (number per genotype in brackets). APOE genotype information was taken from Alzforum database and literature (Supplementary Table S1). * Number of cases included in this study vs total reported cases according to the Alzforum database

The consistent linear correlations between GSEC function readouts and AAOs support the utility of in vitro GSEC activity assays in predicting AAOs for *PSEN2* mutations. Using a leave-one-out cross-validation approach, we estimated biochemical AAOs based on processivity and Aβ40/42 correlative data sets (Figs. 2E and F). Figure 3A shows the AAO variability within carriers and families affected by the same *PSEN2* mutation and contrasts these clinical data (in grey) with biochemically predicted AAO intervals (purple for processivity and green for Aβ40/42 ratio, respectively). The comparison (Fig. 3A-B) revealed significant mismatches between clinical and biochemically predicted AAOs for several mutation carriers at the individual level. Negative mismatch values (AAO—AAO predicted ≤ − 5 years) were observed in carriers of the *PSEN2*-M239T; positive mismatches (AAO—AAO predicted ≥ + 5 years) in carriers of the *PSEN2*- E126 K, S175F, G212V and the M239V mutations; and both positive and negative mismatches in carriers of the *PSEN2*- N141I (Volga) and M239I variants (Supplementary Table S1). We used 5 years as an arbitrary threshold. The variation in clinical AAO for the *PSEN2* L238F variant precluded AAO prediction, though data trends suggest a relatively late AAO (> 65 years).

These predicted AAOs serve as reference values that may help to identify mutation carriers potentially harbouring modulators of symptom onset beyond the *PSEN2* mutation itself. For instance, in the *PSEN2*-N141I (Volga) mutation (AAO_predicted_: 45.7y), the earliest AAO (40y in family R, [31]) shows a − 5.7y mismatch, while mismatch values larger than + 10y and + 20y are observed in 28 and 16 carriers, respectively (AAO ≥ 56 y or ≥ 67y, respectively) (Supplementary Table S1). These negative and positive discrepancies suggest the influence of ‘pathogenic’ and ‘protective’ (genetic and/or environmental) modifiers of onset, respectively. Notably, all 10 members of the ‘KS’ family present with AAOs (AAO average = 66 y [31]) significantly later than predicted, despite the presence of the ApoE4 allele, suggesting the influence of protective factors in this family.

### APP mutation analysis reveals distinct Aβ profiles for disease-linked mutations in APP TMD

Mutations in the *APP* gene represent another cause of early-onset ADAD, with their effects varying based on their location within the protein. Mutations in the extracellular region of the APP_C99_ substrate primarily affect the aggregation propensity of the derived Aβ peptides, potentially accelerating amyloid seeding and plaque formation [2]. In contrast, mutations within the transmembrane domain (TMD) of APP can influence GSEC function during sequential proteolysis, leading to altered Aβ profiles [11].

**Fig. 4:**
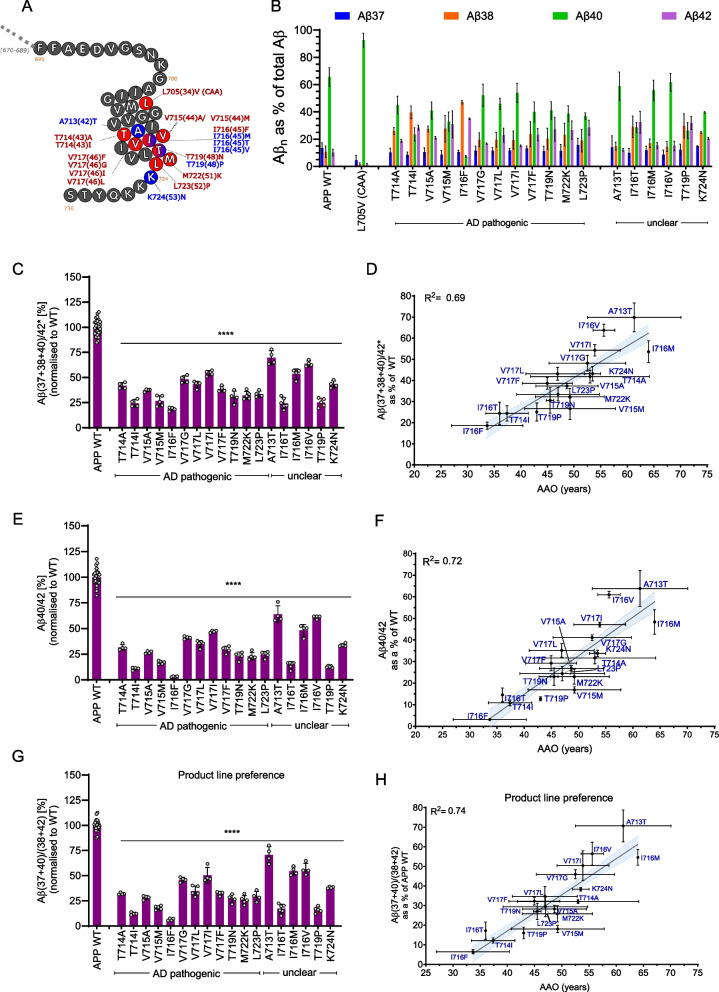
APP TMD mutations alter GSEC processivity and function, paralleling *PSEN1/2* mutation effects. **A** Schematic representation of APP TMD primary structure highlighting residues affected by selected mutations. Color-coding: red: pathogenic and blue: not classified. Mutation positions in *APP* are shown, and the corresponding position in the Aβ sequence is shown in brackets. Pathogenicity information was taken from Alzforum database. **B** Aβ profiles (relative abundance of Aβ37, Aβ38, Aβ40, and Aβ42 peptides relative to total Aβ levels) generated by HEK293T cells expressing WT or mutant APP_C99_ substrates. Aβ43 levels (very low) were excluded (*); Aβ43 levels were measured in at least 3 independent experiments (Supplementary Figure S4). Data presented as mean ± SD, N ≥ 3 independent experiments. **C** Efficiency of the 4 th enzymatic turnover of APP_C99_ (GSEC ‘dysfunction’) quantified by the adapted (*) processivity Aβ(37 + 38 + 40)/42 ratio, normalised to APP WT. Data presented as mean ± SD, N ≥ 3 independent experiments. Statistics: One-way ANOVA with Dunnett's post-hoc test vs WT; ****p < 0.0001, F(18, 77) = 157.5. **D** Correlation analysis between Aβ(37 + 38 + 40)/42* processivity ratio and clinical AAOs for APP TMD mutations. Significant linear correlation found (equation: Y = 1.5 x—34, R^2^ = 0.69) and 95% confidence interval shown as blue area. Error bars represent SD for Aβ ratio (x-axis) and AAO (y-axis). **E** Aβ40/42 ratio data normalised to WT APP. Data presented as mean ± SD, N ≥ 3 independent experiments. Statistics: One-way ANOVA with Dunnett's post-hoc test vs WT; ****p < 0.0001, F(19, 80) = 231.9. **F** Correlation analysis between Aβ40/42 ratio and clinical AAOs for APP TMD mutations. Significant linear correlation found (equation: Y = 1,8*X – 57, R^2^ = 0.72) and 95% confidence interval shown as blue area. Error bars represent SD for Aβ ratio (x-axis) and AAO (y-axis). **G** Product line preference ratio (Aβ(37 + 40)/(38 + 42)) data normalised to WT APP. Data presented as mean ± SD, N ≥ 3 independent experiments. Statistics: One-way ANOVA with Dunnett's post-hoc test vs WT; ****p < 0.0001, F(18, 85) = 286.3. **H** Correlation analysis between Aβ(37 + 40)/(40 + 42) product line preference ratio and AAOs for APP TMD mutations. Significant linear correlation found (equation: Y = 1.8 x—57, R^2^ = 0.74) and 95% confidence interval (blue area). Error bars represent SD for Aβ ratio (x-axis) and AAO (y-axis)

To investigate how mutations in the APP TMD affect GSEC processing of Aβ, we analysed 18 different mutations, classified as ADAD pathogenic or variants of unclear significance. Additionally, we analysed the *APP-L705V* (Piedmont) mutation, which is associated with pure cerebral amyloid angiopathy (CAA) that presents without parenchymal Aβ plaques or tau pathology but with recurrent intracerebral haemorrhage [32] (Table 2, Fig. 4A). We transiently expressed WT and mutant APP_C99_ substrates in WT HEK cells (expressing endogenous human GSEC), collected conditioned media after 30 h and measured secreted Aβ peptides. GSEC processivity was similar between MEF PSEN1 WT [23] and the HEK WT model, allowing us to investigate the effects of APP mutations on Aβ production in the context of normal GSEC function. Aβ profiles derived from tested *APP* mutations (Fig. 4B) revealed consistent patterns among pathogenic and unclear mutations: relative increases in Aβ42 and Aβ38 along with relative decreases in Aβ40. Importantly, WT Aβ peptides are generated from most of these mutant substrates, except for the *APP*- A713T, T714A, and T714I mutations, which affect positions 42 and 43 in Aβ. For the quantification of mutant Aβ42 that is generated from the A713T (A42T) mutation, we developed a specific ELISA-based method (detailed in the methodology section).

Aβ profile analysis also revealed no significant changes in Aβ43 levels for most pathogenic APP mutations, with the exception of T719N, which exhibited a significant increase (Supplementary Figure S4A). Given that Aβ43 levels remained largely unchanged for most mutations and the inclusion of this peptide did not change the processivity Aβ(37 + 38 + 40)/(42 + 43) ratio when comparing it to the Aβ(37 + 38 + 40)/42* ratio (three independent experiments, Supplementary Figure S4B), we opted to exclude this specific peptide from further analysis. It should be noted that for the APP- T714A and T714I mutations, the Aβ43 peptide levels were not measured due to the substantial effort required to develop specific detection methods.

Our analysis revealed that the Aβ(37 + 38 + 40)/42* ratio, referred to as ‘GSEC dysfunction’ for clarity, was consistently decreased by all pathogenic and unclear APP mutations (Fig. 4C). The analysis of the Aβ40/42 ratio showed similar results (Fig. 4E). Consistent with previous reports [9, 33, 34], the GSEC product-line preference (Aβ(37 + 40)/(38 + 42)* ratio, Fig. 4G) showed mutation-induced changes that favour the Aβ42 product-line; these alterations were significant for all pathogenic and unclear *APP* mutations. The analysis of the Aβ38/42 ratio, which informs about the efficiency of the Aβ42 → Aβ38 cleavage, showed no significant changes for the mutant APP substrates, relative to WT APP (Supplementary Figure S5A). This is not surprising given that WT Aβ42 is generated by GSEC from all APP mutations, except for one mutation (A713T, A42T in Aβ). Additionally, both pathogenic and unclear mutations significantly lowered the Aβ37/42 ratio (Supplementary Figure S5B). However, changes in this ratio were overall less pronounced than those observed for the Aβ(37 + 38 + 40)/42*, Aβ40/42, and Aβ(37 + 40)/(38 + 42) ratios.

In contrast, the CAA-linked L705V mutation induced a marked increase in Aβ40 production (92.6% of the total peptides) (Fig. 4B), which translated into a substantial increase in the Aβ(37 + 38 + 40)/42* and Aβ40/42 ratios, compared to the WT APP substrate (Supplementary Figure S5C-D). These findings demonstrate that disease-linked mutations in APP TMD significantly but differentially alter the processing of APP by GSEC and resultant Aβ profiles.

### APP TMD mutation-induced shifts in GSEC function linearly correlate with AAO

We next investigated the relationships between Aβ ratios and disease severity (AAO). We found strong linear correlations between APP mutation-induced changes in the Aβ(37 + 38 + 40)/42*, the Aβ40/42 and the Aβ(37 + 40)/(38 + 42) ratios and AAO; which are described by: Y = 1.5 x – 34 (R^2^ = 0.69) (Fig. 4D), Y = 1.8 x – 57 (R^2^ = 0.72) (Fig. 4F) and Y = 1.8 x – 57 (R^2^ = 0.74) (Fig. 4H), respectively. Furthermore, we found a significant but weaker correlation between the Aβ37/42 ratio and the AAO (Y = 1.5 x – 34, R^2^ = 0.48) (Supplementary Figure S5E). The strength of these correlations provides robust evidence that mutation-induced alterations in GSEC-mediated processing of APP are closely linked to ADAD onset.

### Biochemical prediction of AAOs for APP TMD variants and comparison with clinical AAOs

**Fig. 5:**
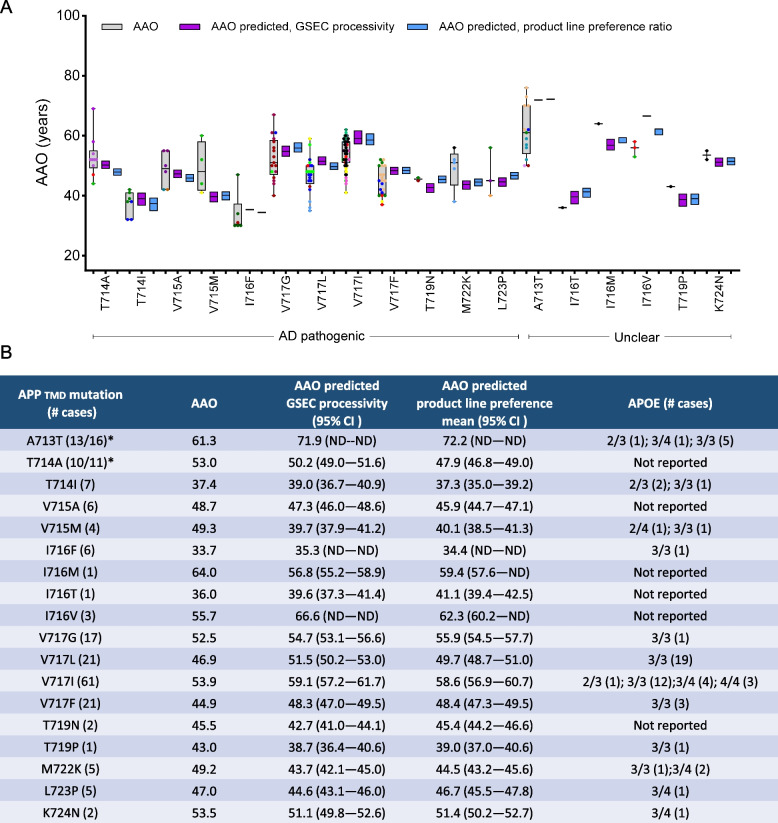
GSEC processivity and product line preference predict AAO in APP TMD variants. **A** Comparison of clinical and predicted AAOs for APP mutations. Grey boxes: clinical AAOs (mean ± SD); coloured dots: individual mutation carriers (each colour represents one family); purple boxes: AAOs predicted based on processivity ratio data (from Fig. 4C,D); blue boxes: AAOs predicted based on product line preference ratio data (from Fig. 4G,H). Predicted AAOs presented as mean ± 95% CI. Mutation classification (AD pathogenic or unclear) according to Alzforum database. **B** Summary table of APP TMD variants that significantly altered Aβ ratios, including: mutation, number of cases, clinical AAO, predicted AAOs (based on GSEC processivity or product line preference ratio data), 95% CI of predicted AAO, and APOE genotypes for reported cases (number per genotype in brackets). APOE genotype information sourced from Alzforum database and literature (Supplementary Table S2). * Number of cases included in this study vs total reported cases according to the Alzforum data base

Using the strongest correlative data (product line preference ratio, Fig. 4H) and the processivity ratio (Fig. 4D), we biochemically predicted mutation-intrinsic AAOs and compared them with clinical AAO averages. This analysis revealed overlapping clinical and predicted AAO intervals for most APP mutations but highlighted significant discrepancies in several cases (Fig. 5A). The comparison showed mismatch values of more than 5 years for the *APP*- T714A (5.1y), I716T (− 5.1y), I716V (− 6.6y) and V715M (9.2y); and more than 10 years for the *APP*- A713T (− 10.9y) (Fig. 5A-B). At the individual level, negative mismatch values larger than 10y were found in carriers of the *APP*- A713T, I716V, V717L, V717I, V717G and V717F variants. Negative mismatches of ~ 20y or more occur in three A713T carriers from different families. Notably, clinical and predicted AAOs align for only one *APP*-A713T family marked in yellow (Fig. 5A). Positive mismatches larger than 10y are seen in one carrier each of the *APP*- T714A, V715M, I716F, V717G, M722K, and L723P variants, while one carrier of the V715M mutation presented mismatches of 20y. These negative and positive mismatches suggest potential influences of pathogenic or protective AAO-modifying factors, respectively. For mutations affecting positions 42 and 43 in Aβ, changes in Aβ aggregation tendency (induced by the amino acid change) may also play a role [20].

### Mechanistic convergence of PSEN1, PSEN2, and APP variants supports an ADAD unifying model

**Fig. 6:**
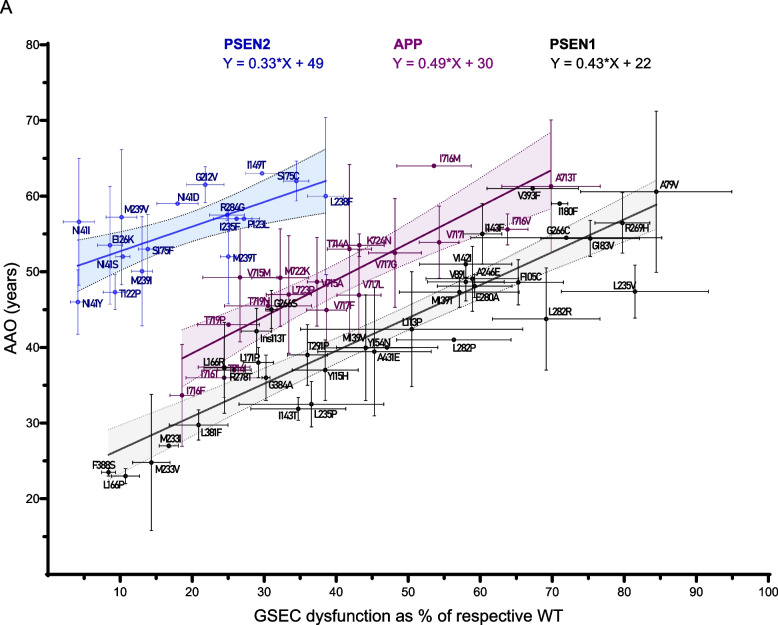
Linear correlations between clinical AAO and GSEC 'dysfunction' for PSEN1, PSEN2, and APP TMD variants. Linear correlations between clinical AAO and GSEC 'dysfunction' assessed by the Aβ (37 + 38 + 40)/(42 + 43) ratio for PSEN1 (black) and PSEN2 (blue), and the Aβ (37 + 38 + 40)/(42)* ratio for APP TMD (purple). The respective linear equations and 95% CIs are shown in the corresponding colours. Error bars represent SD for Aβ ratio (x-axis) and AAO (y-axis). 95% CIs for slopes (m) are PSEN1: 0.36 to 0.51; PSEN2: 0.14 to 0.52; APP: 0.33 to 0.64. 95% CIs for y-intercepts (b): PSEN1: 18 to 26; PSEN2: 45 to 54; APP: 23 to 36

To compare mutation effects across the three causal genes, we plotted the correlation for *PSEN1* (included in Petit et al. 2022 [23] plus ten additional variants, Supplementary Table S3), *PSEN2* and *APP* mutations together for the processivity ratios and AAOs. By plotting Aβ ratios on the x-axis and AAO on the y-axis, we could directly visualize'shifts in AAO'through differences in the y-intercept (b) of the linear correlations (Y = m*X + b, where m is the slope). Our analysis yielded the following linear equations: PSEN1: Y = 0.43*X + 22 (R^2^ = 0.81); PSEN2: Y = 0.33*X + 49 (R^2^ = 0.47), and APP: Y = 0.49*X + 30 (R^2^ = 0.74). The 95% confidence intervals (CI) for the slopes (m) overlap across genes (PSEN1 95% CI: 0.36 to 0.51; PSEN2 95% CI: 0.14 to 0.52 and APP 95% CI: 0.33 to 0.64), indicating a similar relationship between Aβ ratios and AAO (Fig. 6). The similarity in slopes suggests a common underlying mechanism by which alterations in Aβ processing contribute to disease onset across *PSEN1*, *PSEN2*, and *APP* mutations. We note that the broader APP 95% CI might reflect the contribution of additional factors (e.g. mutation-induced changes in Aβ aggregation propensity) to AAO.

The Y-intercepts (b) showed no overlap between PSEN1 and PSEN2 and a partial overlap between PSEN1 and APP (PSEN1 95% CI: 18 to 26; PSEN2 95% CI: 45 to 54 and APP 95% CI: 23 to 36). The distinct y-intercepts (PSEN1: 22 years, PSEN2: 49 years and APP: 30 years) provide quantitative data that weigh the contribution of genetic context to ADAD symptom onset. Compared to *PSEN1* variants, mutations in *PSEN2* and *APP* have a ‘delayed onset’ by 27 years and 8 years, respectively. The combination of similar slopes and different Y-intercepts highlights the complexity of ADAD pathogenesis, where both shared mechanisms and gene-specific factors contribute to the disease timeline.

### Severely dysfunctional and inactivating PSEN1 variants ‘delay’ disease onset

**Fig. 7:**
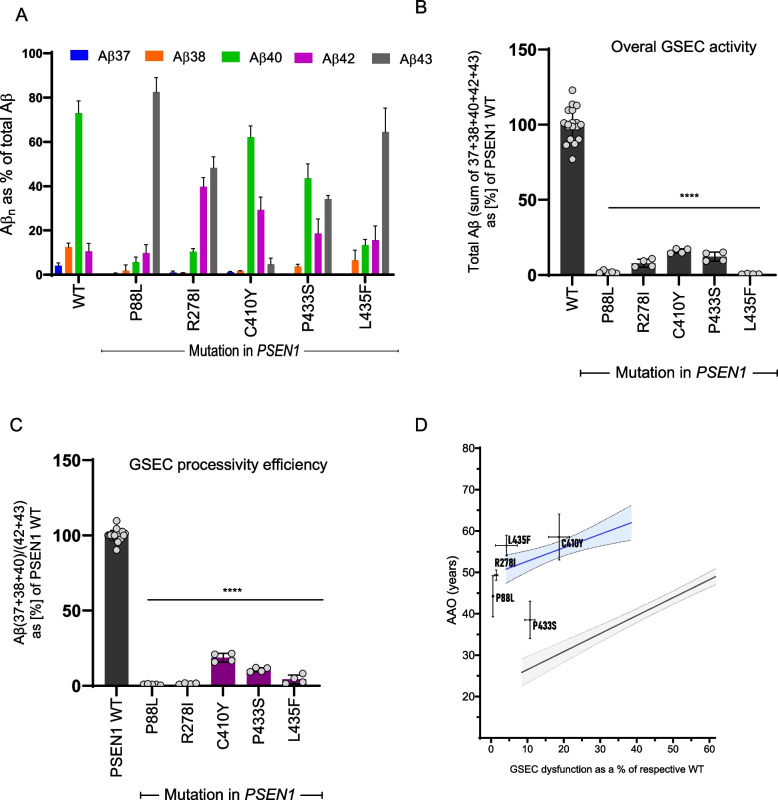
Strong inactivating PSEN1 variants exhibit delayed AAOs, relative to biochemically predicted ones, despite Aβ signatures of pathogenicity. **A** Aβ profiles generated by WT or extremely inactivating PSEN1s. Data presented as mean ± SD, N ≥ 3 independent experiments. **B** Effects of extremely inactivating PSEN1 mutations on the overall GSEC activity, using the sum of the Aβ (37 + 38 + 40 + 42 + 43) as proxy. Data is normalized to PSEN1 WT. Data presented as mean ± SD, N ≥ 3 independent experiments. Statistics: One-way ANOVA with Dunnett's post-hoc test vs WT; ****p < 0.0001, F(5, 33) = 251.9. **C** Efficiency of 4th enzymatic GSEC turnover of APP_C99_ (estimate of GSEC processivity) quantified by the Aβ(37 + 38 + 40)/(42 + 43) ratio. Data is normalised to PSEN1 WT (shown in black). The inactivating mutations are shown in purple. Data presented as mean ± SD, N ≥ 3 independent experiments. Statistical significance determined by one-way ANOVA and Dunnett's post-hoc test compared to PSEN1 WT (*p* < 0.05); ****p < 0.0001, (F(DFn, DFd): F (5, 39) = 2456). **D** Extremely inactivating PSEN1-P88L, R278I, C410Y, P433S and L435F mutations show more than a 20 year delays in AAOs, with the exception of one P433S mutation carrier (Supplementary table S3), and relative to biochemically predicted AAOs

The delayed disease onset in *PSEN2* carriers compared to *PSEN1* carriers, despite similar alterations in Aβ profiles, parallels previous observations by Szaruga et al., 2017 [11] for extremely destabilizing *PSEN1* variants. This previous analysis suggested that severe inactivating mutations, leading to both GSEC partial inactivation and dysfunction, paradoxically attenuate pathogenic impact. To further investigate this hypothesis, we characterized Aβ profiles for all previously reported extremely inactivating PSEN1 mutations (P88L, R278I, C410Y, P433S and L435 F) [11, 17, 23] (Fig. 7A). These profiles exhibit marked reductions in the overall GSEC activity (≥ 85% inactivation, Fig. 7B) and processivity (Fig. 7C), with relative increases in Aβ43 and Aβ42 peptides. Correlation analysis of processivity versus AAO showed that, relative to predictions based on the general PSEN1 correlative data, these mutations are associated with ‘delayed’ clinical onset of more than 20 years for almost all of the cases (except one carrier of the P433S mutation, Fig. 7D and Supplementary table S3). This is despite their strong pathogenic shifts on Aβ profiles towards longer Aβ peptides (Fig. 7A).

## Discussion

The relationship between GSEC dysfunction and ADAD pathogenesis (including onset and progression) has been established through biochemical assessment of *PSEN1* variants [23, 24]. Building on this foundation, we expanded this in vitro approach to examine mutations in the *PSEN2* and *APP* genes.

### Comparative analysis of PSEN1, PSEN2, and APP mutations

We analysed two mutation sets: *i*) 28 *PSEN2* mutations scattered throughout the PSEN primary sequence, classified as pathogenic,'likely pathogenic','variants of unclear significance', and benign (serving as controls) (Table 1), and *ii*) 18 *APP* (TMD) mutations, classified as ADAD pathogenic or variants of unclear significance, including one *APP* (TMD) mutation associated with pure cerebral amyloid angiopathy CAA (L705V) [32]. The APP-TMD mutations (listed in Table 2) were selected based on their potential to alter GSEC-APP/Aβ interactions and thus shift Aβ production [11].

To capture different aspects of GSEC function, we calculated various Aβ ratios, including the processivity Aβ(37 + 38 + 40)/(42 + 43), the product-line preference Aβ(37 + 40)/(38 + 42), the Aβ40/42 and the Aβ37/42 ratios. This analysis revealed that 17 out of 28 *PSEN2* variants significantly and consistently alter all tested Aβ ratios, with the *PSEN2*-A85V and T421M mutations specifically affecting the Aβ37/42 ratio. The fact that the T421M mutation is a benign variant highlights potential limitations of the Aβ37/42 ratio. These overall consistent findings support the pathogenicity of the *PSEN2*- T122P, P123L, E126K, N141D/I/S/Y, I149T, S175C/F, I235F, G212V, L238F, M239I/T/V and R284G mutations. Furthermore, like *PSEN1* mutations, *PSEN2* variants exert their pathogenic effects by impairing the ability of GSEC to efficiently cleave longer Aβ peptides into shorter species (referred to as GSEC dysfunction [10]). However, it is noteworthy that *PSEN2* mutation-driven shifts in Aβ profiles also arise from changes in the GSEC product line preference [6], and this contribution is less evident in *PSEN1* pathogenicity [23].

Our findings revealed linear correlations between both the processivity and Aβ40/42 ratios with AAOs (R^2^ = 0.52 and R^2^ = 0.50, respectively). These correlations for *PSEN2* mutations were notably weaker than those previously observed for *PSEN1* mutations (R^2^ = 0.78, Petit et al*.* 2022 [23]), which could reflect the influence of additional factors (*see below*). Supporting our findings, Liu et al*.* [35] recently reported similar patterns of Aβ production across homologous (sister) *PSEN1* and *PSEN2* variants and their relationship with AAO, using the Aβ42/40 (R^2^ = 0.58) and Aβ37/42 (R^2^ = 0.68) ratios. The independent validation across laboratories and cellular assays supports the notion that PSEN1 and PSEN2 exert pathogenicity and modulate AAO through similar mechanisms (GSEC dysfunction).

For APP-TMD mutations, we observed strong correlations between Aβ ratios and AAO, with the product-line preference Aβ(37 + 40)/(38 + 42) ratio showing an even stronger correlation (R^2^ = 0.74) than the processivity ratio (R^2^ = 0.69). This suggests that *APP* mutations may primarily act by altering the position of the first GSEC cleavage on APP, which selects between the Aβ40 and Aβ42 production pathways.

In contrast to the general trend observed in ADAD-associated mutations, analysis of the *APP*-L705V (Piedmont) mutation revealed a profile enriched in Aβ40. This is consistent with prior pathology studies linking Aβ40 to cerebral amyloid angiopathy [36], and suggests a different pathogenic mechanism for cerebral amyloid angiopathy compared to ADAD. While the underlying basis for the marked increase in Aβ40 remains to be determined, the strong reduction in both Aβ38 and Aβ42 suggests that a shift in GSEC product-line preference, opposite to that identified in ADAD-linked mutations, could be at play. In any case, this finding highlights the potential for different Aβ profiles to drive distinct pathological outcomes.

### A unified model of ADAD pathogenesis and AAO

Comparative analysis of the processivity-AAO correlations across *PSEN1*, *PSEN2*, and *APP* (TMD) mutations revealed parallel (similar slopes, m) but shifted lines (shifted Y-intercepts, b), supporting a shared pathogenic mechanism and gene-specific effects. While these correlations are statistically significant, they explain only part of the observed AAO variability (R^2^ ranging from 0.50 to 0.78), emphasizing the complex nature of ADAD pathogenesis and suggesting the potential contribution of exogenous factors. Specifically, our results support a model where shifts in the short-to-long Aβ peptide ratio, whether through direct changes in GSEC processivity or shifts in GSEC product-line preference, are central to disease onset and likely progression (given the findings reported by Schultz et al. [24]). The linear patterns (greater mutation-induced impairments in Aβ processing correspond to earlier AAO) imply a dose–response relationship between the degree of shift in Aβ profiles and disease severity, albeit modulated by gene-specific factors (distinct Y-intercepts). Therefore, the quantitative framework established here provides a baseline for systematic investigations into how downstream mechanisms – including tau pathology, neuroinflammation, and altered proteostasis, among others – interact with initial Aβ changes to influence disease onset and progression. Particularly, cases where clinical onset deviates significantly from the predicted AAO provide opportunities to study these complex interactions.

The weaker correlative data for PSEN2 and APP, compared to PSEN1 mutation (R^2^ = 0.78, Petit et al. 2022 [23]), could be at least in part explained by the modulation of AAO through genetic and/or environmental factors. Notably, our analysis reveals a spectrum of mutation effects, including subtle yet significant shifts in Aβ ratios that are associated with variants linked to incomplete penetrance or considered as risk factors (e.g., *PSEN1*-A79 V, *PSEN2*-L238 F and *APP*-A713 T [37, 38, 39, 40]. We speculate that, while Aβ profile shifts are primary, the very mild effects of these mutations, and their respective PSEN sister mutations (*PSEN2*-A85V, *PSEN1*-L232F), could situate them at the threshold for pathogenicity. This situation might confer a high susceptibility to modulation by exogenous factors, potentially explaining an incomplete penetrance. AAO could also be influenced by other mechanisms, such as stochastic cellular processes, epigenetic changes and genetic instability (*see below*).

### Gene-specific contributions to disease onset

The distinct Y-intercepts (b) observed (PSEN1: 22 years, PSEN2: 49 years and APP: 30 years) provide, for the first time, quantitative data that weigh the contribution of genetic context to ADAD onset. Compared to *PSEN1* variants, *PSEN2* and *APP* mutations delay onset by 27 years and 8 years, respectively. These differences likely reflect underlying biological variations among the three genes, possibly arising from differences in protein expression levels, functional levels [5] and cellular/tissue localization [6]. PSEN2-type GSECs account for only 16%–35% of APP processing in the brain [41]. This reduced contribution to APP metabolism may explain its later onset and may arise from their restricted cellular and brain tissue distribution [6, 42] and/or their lower catalytic efficiency [5], both compared to PSEN1 complexes [6]. Supporting this (Aβ-AAO) dosage-dependent mechanism, the analysis of the extremely inactivating PSEN1- P88L, R278I, C410Y, P433S and L435F variants shows that reduced pathogenic allele contributions to APP processing in the brain translate into delayed onset.

Unlike *PSEN1/2* mutations, APP mutations influence Aβ processing by all types of GSEC complexes. We hypothesize that this 'global impact' results in an intermediate effect on AAO, positioning *APP* mutations between the earlier-onset *PSEN1* and later-onset linked to *PSEN2* in terms of AAO. The relatively smaller shift (8 years) for APP mutations and stronger correlations resembling *PSEN1* findings fit well with the notion that PSEN1-GSECs are the main contributors to amyloid metabolism in the brain.

### Biochemical analysis of ADAD-causality: implications for identification of AAO modifying factors

While correlative evidence cannot establish causation, our analysis puts forward a unifying framework for understanding ADAD pathogenesis that incorporates both shared mechanisms and gene-specific variations in AAO. Our biochemical measurements provide reference points against which to evaluate clinical AAO variability and potentially possible therapeutic effects of disease-modifying treatments (*see below*). Importantly, this framework generates predicted AAOs for PSEN1/2 and APP TMD variants, enabling the systematic identification of carriers whose actual onset significantly deviates from biochemical predictions. Until now, such identification of AAO modifiers has been limited to the relatively large PAISA population (PSEN1-E280A carriers) in Colombia [43], where studies identified the Christchurch *APOE* mutation [44] and Reelin [45] as exogenous factors that influence disease onset timing (modifiers of AAO) in *PSEN1* carriers through mechanisms beyond initial Aβ changes. Such findings are of paramount importance in enhancing our understanding of disease mechanisms downstream of Aβ and serve as valuable starting points for translational research. Our approach extends this capability across different *PSEN1/2* and *APP* variants, providing new opportunities to investigate how genetic background and downstream mechanisms – including tau pathology, neuroinflammation and altered proteostasis – modulate the relationship between GSEC dysfunction and clinical onset.

The higher variability in AAOs among PSEN2 and APP mutation carriers may indicate an increased susceptibility to genetic and/or environmental modifiers relative to PSEN1. Our quantitative analysis reveals positive and negative mismatches (AAO – AAO predicted ≥ ± 5 years) for several *PSEN2* and *APP* mutation carriers. Examining the mutant PSEN2 (N141I (Volga) and M239V) and APP (V717G, V717L, V717I, V717F and A713T) cohorts shows that a large proportion of carriers presented with dementia later than expected for PSEN2, and earlier than expected for APP, based on biochemical AAOs. While it could result from genetic/environmental AAO modifiers, other mechanisms could also operate.

Evidence for such mechanisms comes from transcript analysis studies comparing PSEN1 and PSEN2 carriers. While PSEN1 variant carriers show balanced expression of WT and mutant alleles (pathogenic transcripts ~ 42–51%), PSEN2 carriers exhibit decreased stability of the variant allele (only 35–37% pathogenic transcripts) [46]. In addition, PSEN2 carriers show more heterogeneous transcript populations, with only ~ 62% encoding full-length protein. Notably, two out of three N141I (Volga) carriers showed prevalent exon 6 skipping, leading to premature termination codons and partial'silencing'of the mutant allele, potentially explaining their delayed onset.

Somatic APP gene recombination has been shown to occur mosaically in neurons in an age-dependent manner [47], increasing APP expression and, ultimately, Aβ levels. A variable APP copy number in neurons from sporadic AD patients has been reported and suggested to contribute to AD [48]. In ADAD, such recombination (if happening) could amplify mutant APP expression, potentially explaining the earlier-than-predicted onset in APP mutation carriers.

In conclusion, the comparison of clinical and predicted AAOs at the individual/family levels may guide future investigations into potential modifiers of AAO and/or other mechanisms modulating the pathogenic contribution of *PSEN1*, *PSEN2* and *APP* causal genes.

### Biochemical analysis of ADAD-causality: implications for therapeutics

Our quantitative findings have direct implications for therapeutic development in ADAD. The slope suggests that even small shifts in Aβ profiles could lead to significant delays in AAO, potentially offering a therapeutic target for delaying disease onset. Specifically, a slope of approximately 0.43 (observed for PSEN1) indicates that for every positive 1% shift in the Aβ profile, there is a corresponding 0.43-year delay in AAO. This suggests that even a modest 12% shift in Aβ profile could lead to a 5-year delay in AAO. Thus, enhancing GSEC processivity (*i.e*., correcting the mutation-induced shift in Aβ profile) could be an effective therapeutic approach not only for the different genetic forms of ADAD but potentially also in the most common sporadic AD [49]. GSEC modulators (GSMs [50]) bind to the extracellular GSEC-Aβ interface [51], activating Aβ processing and shifting profiles towards shorter Aβ peptides while preserving the overall GSEC activity, which has essential roles in cellular homeostasis. The potential use of GSMs is backed by positive safety outcomes from a Phase 1 trial with a second-generation GSM (https://www.alzforum.org/news/conference-coverage/second-generation-g-secretase-modulator-heads-phase-2). However, special considerations are needed for mutations in APP affecting the aggregation propensities of Aβ profiles, a pathogenic mechanism that operates downstream of APP/Aβ processing.

In addition, the biochemical data highlights gene-specific baseline differences in AAO. While similar slopes in the AAO-Aβ correlations indicate comparable absolute delays across genes, the timing of these effects varies with mutation type, as reflected by distinct Y-intercepts. Since PSEN2 and APP mutation carriers experience delays later in life than PSEN1 carriers, therapeutic strategies may require earlier initiation in PSEN1 carriers to achieve optimal benefits.

Our findings with the strongly inactivating PSEN1 variants support the selective silencing of the pathogenic allele in therapeutic settings. While this approach is restricted to familial AD, GSEC modulators, acting as GSEC stabilizers, could have broader therapeutic value, extending to sporadic AD [49]. In this more common form of AD, impaired Aβ peptide clearance leads to progressive accumulation of longer (versus shorter) Aβ peptides, which in turn promotes (as in ADAD) the assembly of toxic Aβs and downstream pathogenic cascades. GSEC stabilizers would prevent these pathogenic cascades by reducing the production of longer Aβ peptides, thereby limiting their accumulation even when brain clearance is compromised in sporadic AD.

In conclusion, by linking shifts in APP/Aβ processing to symptom onset, this analysis lays the groundwork for future research focused on mechanisms modulating AAO broadly in ADAD, including those downstream of Aβ, and supports the therapeutic development of strategies that modulate Aβ generation with implications for sporadic AD.

## Limitations

Our study also has some limitations. First, our analyses were conducted in cell culture models, which do not fully recapitulate the complexity of the mutation heterozygous human brain, where both mutant and WT (*PSEN1, PSEN2*) alleles contribute to APP/Aβ processing. Future studies using more complex patient-derived cellular or animal models carrying these mutations in heterozygous conditions could provide further insights. Second, our conclusions, primarily based on correlative evidence, cannot fully establish causation. Third, while our biochemical approach provides valuable insights, it addresses only one facet of the complex ADAD pathophysiology. However, our analysis is valuable because it provides a framework to investigate interactions between amyloid and downstream pathological processes. Finally, our study focuses on APP as a GSEC substrate. While the impact of *PSEN1/PSEN2* mutations on other GSEC substrates and contribution to ADAD remain to be elucidated, the fact that APP variants alone cause AD indicates that alterations in the processing of other GSEC substrates, while potentially contributing, are not essential for AD pathogenesis. These alterations may, however, explain additional clinical features observed in PSEN1/2 mutation carriers.

### Methodology

#### Antibodies and reagents

The following antibodies were used in western blot analyses: mouse anti-NCT (9C3) kindly provided by Prof. Wim Annaert; anti-human PSEN1-CTF (MAB5232) purchased from Merck Millipore; anti-human PSEN2-CTF (ab51249) purchased from Abcam and anti-human PEN2 (DGG8) purchased from Cell Signaling. Horse radish peroxidase (HRP)-conjugated anti-mouse (#1,721,011) and anti-rabbit IgG (#1,721,019) purchased from Bio-Rad and anti-rat IgG (#61–9520) purchased from Thermo Fisher. The following antibodies were used in the MesoScale Discovery (MSD) multi-spot Aβ ELISA, obtained through collaboration with Janssen Pharmaceutica NV (Beerse, Belgium):the JRD/Aβ37/3 for Aβ37, JRF AB038 for Aβ38, JRF/cAb40/28 for Aβ40 and JRF/cAb42/26 for Aβ42 as capture antibodies. As detection antibody, we used the 6E10 antibody (Biolegend), raised against the N terminus of Aβ (1–16 amino acids), conjugated with MSD GOLD Sulfo-Tag NHS-Ester. The anti-Aβ43 rabbit IgG (capture antibody) and anti-Aβ (N) (82E1) mouse IgG Fab’ (detection antibody) were both supplied with the ELISA kit for Aβ43 (IBL).

#### Generation of stable cell lines expressing WT or mutant GSEC complexes

We transduced* psen1*^*−/−*^*psen2*^*−/−*^ (dKO) mouse embryonic fibroblasts (MEFs) with retroviruses expressing human WT PSEN1, WT PSEN2 or mutant PSEN2s. The retroviral expression system (Clontech) was used as described previously [9]. Briefly, HEK293T17 cells were co-transfected with pMSCVpuro encoding WT or mutant human PSEN2 (or PSEN1) and a helper packaging vector. Retroviral particles were harvested 48 h post transfection, filtered (0.45 µm pore size filter) and used to transduce the dKO MEFs cultured in Dulbecco’s Modified Eagle’s Medium (DMEM)/F- 12 supplemented with 10% fetal bovine serum (FBS). Cells stably expressing the human PSEN2 proteins were selected with puromycin (5 µg/ml) and maintained in culturing medium supplemented with 3 µg/ml puromycin. To confirm PSEN2 expression and reconstitution of active GSEC complexes, we prepared and solubilized membranes in 1% CHAPSO, 28 mM PIPES pH 7.4, 210 mM NaCl, 280 mM sucrose, 1.5 mM EGTA pH 8 and 1 × complete protein inhibitor mix (Roche) buffer. Proteins were resolved on 4–12% Bis–Tris NuPAGE gels (ThermoScientific) and transferred to nitrocellulose membranes. We used antibodies against PSEN1/2-CTF, PEN- 2, and Nicastrin to verify complex formation. Western Lightning Plus-ECL Enhanced Chemiluminescence Substrate (Perkin Elmer) and Fuji imager was performed to visualized the gels.

#### Expression of APP_C99_ in MEF cell lines

For cell-based activity assays with MEF PSEN1/2 WT or mutants, cells were plated at a density of 15 000 cells/well in 96 well plate. 4 h after plating, cells were transduced with an adenoviral vector encoding human APP_C99_ and green fluorescence protein (GFP) expressed under a different promoter to control for transduction efficiency. 16 h after transduction the medium was changed to low-serum medium (DMEM + 0.2% FBS). After 30 h of incubation, the conditioned medium was collected for Aβ analysis.

#### Expression of WT and mutant APP_C99_ in HEK cells and analysis of GSEC activity

The mammalian expression pSG5-APP_C99_− 3xFLAG construct was used for site-directed mutagenesis to generate APP mutations. To determine the effects of WT or mutant APP, HEK293T cells were plated at a density of 30 000 cells/well in 96-well plate. The next day, cultures were transiently transfected with the different WT/mutant constructs using 1 mg/ml polyethyleneimine (PEI) solution with a DNA:PEI ratio of 1:3. 24 h after transfection, the medium was changed from DMEM + 10% FBS to DMEM + 2% FBS and then collected 30 h after for Aβ analysis as previously described for the analysis of PSEN2 mutants.

#### Aβ peptide quantification

We used Multi-Spot 96-well MSD ELISA plates to quantify Aβ37, Aβ38, Aβ40, and Aβ42 peptides. The plates were coated with specific antibodies for each Aβ species. Non-specific protein binding to the plates was blocked with 150 μl/well blocking buffer (PBS supplemented with 0.1% casein) for at least 1.5 h at room temperature. After blocking, we added samples or standards mixed (1:1) with Sulfo-Tag 6E10 detection antibody diluted in blocking buffer. After overnight incubation, plates were washed 5 times with PBS, 0.05% Tween and the signals measured using a Sector Imager 6000 (Meso Scale Discovery). For Aβ43 quantification, we used the human Amyloid β (1–43) (FL) assay kit from IBL, following the manufacturer's protocol.

To measure total Aβ levels, we used single-spot 96-well MSD ELISA plates coated with 50 µL/well of 4G8 antibody (SIG- 39220, purchased from BioLegend) diluted at 3 µg/ml in PBS (overnight incubation). The next day, plates were washed 5 times with PBS, 0.05% Tween buffer and blocked. Samples or standards were added mixed with 6E10 detection antibody and incubated overnight. After overnight incubation, plates were washed 5 times with PBS, 0.05% Tween and the signals measured using a Sector Imager 6000 (Meso Scale Discovery). To measure intracellular Aβ levels, we first rinsed the 96 well plates with PBS and we added 50 µL of RIPA buffer with proteinase inhibitors (PI) and incubated for 1 h on ice. We then collected the samples and centrifuged at 14 000 g for 15 min. To quantify Aβ37, Aβ38, Aβ40, and Aβ42 levels, we used Multi-Spot 96-well MSD ELISA/or 4G8 ELISA as described before.

For the Aβ profile analysis of the mutant APP_C99_A713T substrate (mutation located at position 42 in Aβ), we used the following synthetic mutant peptide as standard for the quantification of Aβ42: DAEFRHDSGYEVHHQKLVFFAEDVGSNKGAIIGLMVGGVVIT. Importantly, the total levels of WT and mutant APP- A713T peptides were set at equal concentrations using the 4G8 ELISA.

### Data analysis

All statistical analyses were performed using GraphPad Prism. We calculated various Aβ ratios, including the processivity ratio Aβ(37 + 38 + 40)/(42 + 43), the product-line preference ratio Aβ(37 + 40)/(38 + 42), the Aβ40/42 ratio or the Aβ37/42 ratio. For PSEN2/APP mutations, we compared these ratios to WT using one-way ANOVA with Dunnett's post-hoc test to establish the significance of the changes between groups. P value < 0.05 was used as a pre-determined threshold for statistical significance. We performed linear regression analysis to examine correlations between Aβ ratios and age of onset (AAO) for each group of mutations (PSEN2 and APP) and determine R^2^ (goodness of fit) and P values. All statistical analyses are described in the figure legends.

## Supplementary Information

Supplementary Material 1.

Supplementary Material 2.

## Data Availability

The data supporting the findings of this study are available from the corresponding authors upon request.

## Abbreviations

AAO: Age at symptom Onset
Aβ: Amyloid-beta
AD: Alzheimer's disease
ADAD: Autosomal Dominant Alzheimer's Disease
AICD: Amyloid precursor protein intracellular soluble domain
ANOVA: Analysis of variance
ApoE: Apolipoprotein E
APH1: Anterior Pharynx Defective 1
APP: Amyloid Precursor Protein
BACE 1: β-Secretase 1
CAA: Cerebral amyloid angiopathy
CTF: C-terminal fragment
DKO: Double Knock-Out
DMEM: Dulbecco’s Modified Eagle Medium
EGTA: Egtazic acid
ELISA: Enzyme-linked Immunosorbent Assay.
FBS: Fetal Bovine Serum
GFP: Green fluorescent protein
GSM: Gamma-secretase modulators
GSEC: γ-Secretase complex
HEK: Human Embryonic Kidney cells
HRP: Horse radish peroxidase
MEF: Mouse Embryonic Fibroblasts
MSD: MesoScale Discovery
NCT: Nicastrin
PI: Proteinase Inhibitors
PEI: Polyethyleneimine
PEN2: Presenilin enhancer 2
PSEN: Presenilin
TMD: Transmembrane domain
WT: Wild Type
