# Supplementary material for: Spectrum of γ-Secretase dysfunction as a unifying predictor of ADAD age at onset across PSEN1, PSEN2 and APP causal genes
Source: Mol Neurodegener. 2025 Apr 26;20:48. doi: 10.1186/s13024-025-00832-1 (PMC12032737; doi:10.1186/s13024-025-00832-1)
Supplement: Supplementary file 1 — Supplementary Material 1. [file 13024_2025_832_MOESM1_ESM.docx]

**SUPPLEMENTARY FIGURE LEGENDS AND TABLE LEGENDS**

**Figure S1: Rescue of WT/mutant GSEC expression in DKO MEFs**

Detergent-extracted membrane proteins from WT and mutant (indicated) cell lines were analysed in SDS-PAGE/western blot. GSEC levels were rescued in *psen1^-/-^ psen2^-/-^* mouse embryonic fibroblasts (DKO MEFs) by stably expressing WT/mutant human PSEN1 or PSEN2. The presence of mature, glycosylated NCTSTN, PSENEN and PSEN C-terminal fragments (PSEN1-CTF or PSEN2-CTF) demonstrate the reconstitution of mature GSECs. PSEN1-CTF levels are shown for the DKO MEF expressing WT PSEN1. Arrowheads indicate the position of the molecular weight markers.

**Figure S2: Characterization of extracellular vs intracellular pool for PSEN2 mutants and reduced Aβ43 levels in PSEN2 mutants.**

1. Characterization of extracellular and intracellular Aβ pools generated by MEFs expressing WT or mutant PSEN2s. All pathogenic and likely pathogenic PSEN2 variants were analysed. Total Aβ pool was calculated as the sum of Aβ37, Aβ38, Aβ40, and Aβ42 peptides measured in both conditioned medium (extracellular) and total cellular lysates (intracellular). Extracellular (blue) and intracellular (orange) pools are shown as percentages. Data plotted as mean ± SD, N ≥ 3 independent experiments.
2. Characterization of extracellular and intracellular Aβ pools generated by MEFs expressing WT or mutant PSEN2s. All the rest of ‘Unclear/Not classified’ PSEN2 variants that showed significant changes in the ratios compared to WT PSEN2 were analysed. Total Aβ pools were calculated with 4G8 ELISA, which detects total Aβ levels, measured in both conditioned medium (extracellular) and total cellular lysates (intracellular). Extracellular (blue) and intracellular (orange) pools are shown as percentages. Data plotted as mean ± SD, N ≥ 3 independent experiments. WT PSEN1 and WT PSEN2 were also included since a different ELISA was performed.
3. Aβ 43/(37+40) ratio normalised to PSEN2 WT (in bold and different filled pattern). PSEN1 WT (blue), PSEN2 and analysed variants are shown in purple and benign variants (control) in grey. Variants are classified according to Alzforum database in: ‘Pathogenic’, ‘Likely pathogenic’, ‘Unclear/Not classified’ and ‘Benign’. Data is presented as mean ± SD, N ≥ 3 independent experiments. One-way ANOVA followed by Dunnett’s post-hoc test with comparison to PSEN2 WT was used to determine statistical significance (p < 0.05); ****p < 0.0001, (F(DFn, DFd): F (29, 152) = 40.84)

**Figure S3: Extra correlations for PSEN2 mutants.**

1. Product line preference Aβ (37+40+43)/(38+42) ratio normalised to PSEN2 WT (in bold and different filled pattern). PSEN1 WT (blue), PSEN2 and analysed variants are shown in purple and benign variants (control) in grey. Variants are classified according to Alzforum database in: ‘Pathogenic’, ‘Likely pathogenic’, ‘Unclear/Not classified’ and ‘Benign’. Data is presented as mean ± SD, N ≥ 3 independent experiments. One-way ANOVA followed by Dunnett’s post-hoc test with comparison to PSEN2 WT was used to determine statistical significance (p < 0.05); ****p < 0.0001, (F(DFn, DFd): F (29, 171) = 52.05)
2. Correlation analysis between the product line preference Aβ (37+40+43)/(38+42) ratio (normalised to PSEN2 WT) and the AAO. This analysis includes all PSEN2 variants showing significant differences compare to PSEN2 WT in the Aβ (37+40+43)/(38+42) ratio. Significant correlation found (equation: Y = 1.8 x – 76, R^2^=0.43). 95% CI is shown as blue area. Error bars represent SD for Aβ ratio (x-axis) and AAO (y-axis).
3. Aβ37/42 ratio normalised to PSEN2 WT (in bold and different filled pattern). PSEN1 WT (in blue), PSEN2 WT and analysed variants are shown in purple and benign variants (control) in light grey. Variants are classified according to Alzforum database in: ‘Pathogenic’, ‘Likely pathogenic’, ‘Unclear/Not classified’ and ‘Benign’. Data is presented as mean ± SD, N ≥ 3 independent experiments. One-way ANOVA followed by Dunnett’s post-hoc test with comparison to PSEN2 WT was used to determine statistical significance (p < 0.05); ****p < 0.0001, (F(DFn, DFd): F (29, 161) = 25.34).
4. Correlation analysis between the Aβ37/42 ratio (normalised to PSEN2 WT) and the AAO. This analysis includes all PSEN2 variants showing significant differences compare to PSEN2 in the Aβ37/42 ratio. Significant correlation found (equation: Y = 1,8*X – 75, R^2^=0.21). 95% confidence interval shown as light blue area. Error bars represent SD for Aβ ratio (x-axis) and AAO (y-axis).
5. Aβ38/42 ratio normalised to PSEN2 WT (in bold and different filled pattern). PSEN1 WT (in blue), PSEN2 WT and analysed variants are shown in purple and benign variants (control) in light grey. Variants are classified according to Alzforum database in: ‘Pathogenic’, ‘Likely pathogenic’, ‘Unclear/Not classified’ and ‘Benign’. Data is presented as mean ± SD, N ≥ 3 independent experiments. One-way ANOVA followed by Dunnett’s post-hoc test with comparison to PSEN2 WT was used to determine statistical significance (p < 0.05); ****p < 0.0001, (F(DFn, DFd): F (29, 172) = 29.67).
6. Correlation analysis between the Aβ38/42 ratio (normalised to PSEN2 WT) and the AAO. This analysis includes all PSEN2 variants showing significant differences compare to PSEN2 in the Aβ38/42 ratio. Significant correlation found (equation: Y = 0,99*X – 38, R^2^=0.21). 95% confidence interval shown as light blue area. Error bars represent SD for Aβ ratio (x-axis) and AAO (y-axis).

**Figure S4: Quantification of Aβ43 peptide for APP mutants.**

1. Quantification of Aβ43 for APP. The amount of Aβ43 is presented as a percentage of total Aβ levels (sum of Aβ37, Aβ38, Aβ40, Aβ42 and Aβ43). T714A and T714I are not included in the measurements since the mutations are located in the position of Aβ43 and that might interfere with the epitope used in the ELISA. APP mutations are divided in: ‘pathogenic’ or ‘unclear’. Statistical analysis using ROUT was performed to exclude outliers from the measurements (Q=1). Data is presented as mean ± SD, N ≥ 3 independent experiments. One-way ANOVA followed by Dunnett’s post-hoc test with comparison to APP wild type was used to determine statistical significance (p < 0.05); ****p < 0.0001, F(16, 46) = 1,864.
2. GSEC processivity data estimated with the Aβ(37+38+40)/(42+43) or Aβ(37+38+40)/(42) ratios, normalised to APP WT, presented in grey and purple, respectively. Data is presented as mean ± SD, N ≥ 3 independent experiments. APP mutations are divided in: ‘pathogenic’ or ‘unclear’. Multiple unpaired T-test followed by Bonferroni-Dunn method to correct for multiple comparisons was used to determine statistical significance (p< 0.05). No significant differences are observed in any of the mutants between these ratios.

**Figure S5: Extra correlations for APP mutants.**

1. Aβ38/42 ratio normalised to APP WT. Variants are classified according to Alzforum database in: ‘pathogenic’ or ‘unclear’. Data is presented as mean ± SD, N ≥ 3 independent experiments. One-way ANOVA followed by Dunnett’s post-hoc test with comparison to APP wild type was used to determine statistical significance (p < 0.05); ****p < 0.0001, (F(DFn, DFd): F (19, 88) = 1,748).
2. Aβ37/42 ratio normalised to APP WT. Variants are classified according to Alzforum database in: ‘pathogenic’ or ‘unclear’. Data is presented as mean ± SD, N ≥ 3 independent experiments One-way ANOVA followed by Dunnett’s post-hoc test with comparison to APP wild type was used to determine statistical significance (p < 0.05); ****p < 0.0001, (F(DFn, DFd): F (19, 88) = 32,46).
3. Efficiency of 4th enzymatic GSEC turnover of APP_C99_ (estimate of GSEC processivity) quantified by the Aβ(37+38+40)/42* ratio for L705V mutation; data normalised to WT. Data is presented as mean ± SD, N ≥ 3 independent experiments. Unpaired two-tailed T test with comparison to wild type was used to determine statistical significance (p < 0.05); ****p < 0.0001, (t= 8,48, df= 20)
4. Aβ 40/42 ratio normalised to WT for the L705V mutation. Data is presented as mean ± SD, N ≥ 3 independent experiments. Unpaired two-tailed T test with comparison to wild type was used to determine statistical significance (p < 0.05); ****p < 0.0001, (t= 10.66, df= 20).
5. Correlation analysis between the Aβ37/42 ratio (normalised to APP WT) and the AAO. Significant correlation found (equation: Y = 1,5*X – 34, R^2^=0.48). 95% confidence interval shown as light blue area. Error bars represent SD for Aβ ratio (x-axis) and AAO (y-axis).

**Supplementary table legends**

**Table S1. Reported AAOs for all studied PSEN2 mutations.** Clinical AAOs; AAO averages and standard deviation; predicted AAOs based on the processivity Aβ (37+38+40)/(42+43) and Aβ40/42 ratios; deviation of clinical and predicted AAOs, and APOE genotypes are shown. Family names, when described, are included in brackets next to the respective AAOs. Families carrying the same mutation are separated by lines.

**Table S2. Reported AAOs for all studied APP mutations.** Clinical AAOs; AAO averages and standard deviation; predicted AAOs based on the processivity Aβ (37+38+40)/(42) and Aβ (37+40)/(38+42) ratios; deviation of clinical and predicted AAOs; and APOE genotypes are shown. Family names, when described, are included in brackets next to the respective AAOs. Families carrying the same mutation are separated by lines.

**Table S3. Reported AAOs for extra PSEN1 mutations included in the correlation (apart from the included in** (1)**) and inactivating PSEN1 mutations.** Clinical AAOs; AAO averages and standard deviation; predicted AAOs based on the processivity Aβ (37+38+40)/(42+43); deviation of clinical and predicted AAOs, and APOE genotypes are shown. Families carrying the same mutation are separated by lines.

(2–12)

| **Supplementary Table S1** | | | | | | | | | |
| --- | --- | --- | --- | --- | --- | --- | --- | --- | --- |
| **Mutation in  PSEN2** | **AAO cases (family)** | **Mean  AAO** | **SD** | **AAO predicted  GSEC processivity** | **AAO - AAO predicted  (GSEC processivity)** | **AAO predicted  Aβ40/42 ratio** | **AAO- AAO predicted  (Aβ40/42 ratio)** | **APOE** | **References** |
| **A85V.** | 60,0 | 61,5 | 6,8 | / | / | / | **/** | APOE 3/3 | Piscopo et al., 2008 |
|  | 60,0 |  |  |  |  |  |  | APOE 3/4 |  |
|  | 55,0 |  |  |  |  |  |  | APOE 3/4 |  |
|  | 71,0 |  |  |  |  |  |  | APOE 3/4 |  |
| **T122P** | 46,0 | 47,3 | 2,3 | 49,7 | -3,7 | 49,7 | -3,7 | Not reported | Finckh et al., 2000 |
|  | 50,0 |  |  |  | 0,3 |  | 0,3 | Not reported | Finckh et al., 2005 |
|  | 46,0 |  |  |  | -3,7 |  | -3,7 | APOE 3/4 | Lanoiselée et al., 2017 |
| **P123L** | 57,0 | 57,0 | / | 61,2 | -4,2 | 62,9 | -5,9 | Not reported | Xia et al., 2015 |
| **E126K** | 48,0 | 53,5 | 7,8 | 48,4 | -0,4 | 48,9 | -0,9 | Not reported | Muller et al., 2014 |
|  | 59,0 |  |  |  | 10,6 |  | 10,1 | Not reported |  |
| **S130L** | 65,0 | 65,2 | 10,3 | / | / | / | / | Not reported | Sorbi et al., 2002 |
|  | 81,0 |  |  |  |  |  |  | Not reported | Tomaino et al., 2007 |
|  | 77,0 |  |  |  |  |  |  | Not reported | Lohmann et al., 2012 |
|  | 52,0 |  |  |  |  |  |  | APOE 3/3 | Wojtas et al., 2012 |
|  | 61,0 |  |  |  |  |  |  | Not reported | Sassi et al., 2014 |
|  | 73,0 |  |  |  |  |  |  | Not reported | Schulte et al., 2015 |
|  | 51,0 |  |  |  |  |  |  | Not reported | Nicolas et al., 2015 |
|  | 62,0 |  |  |  |  |  |  | Not reported | Nicolas et al., 2015 |
|  | 65,0 |  |  |  |  |  |  | APOE 3/3 | Sala Frigerio et al., 2015 |
| **N141D** | 59,0 | 59,0 | / | 54,7 | 4,3 | 54,9 | 4,1 | APOE 3/3 | Wang et al., 2019 |
| **N141I** | 57,0 | 56,6 | 8,4 | 45,7 | 11,3 | 45,0 | 12,0 | APOE 3/3 | Levy-Lahad et al.,1995 |
|  | 58,0 |  |  |  | 12,3 |  | 13,0 | APOE 3/3 |  |
|  | 62,0 (BE) |  |  |  | 16,3 |  | 17,0 | APOE 3/3 |  |
|  | 61,0 |  |  |  | 15,3 |  | 16,0 | APOE 3/? |  |
|  | 62,0 |  |  |  | 16,3 |  | 17,0 | APOE 3/? |  |
|  | 57,0 |  |  |  | 11,3 |  | 12,0 | APOE 3/? |  |
|  | 51,0 |  |  |  | 5,3 |  | 6,0 | APOE 3/? |  |
|  | 52,0 |  |  |  | 6,3 |  | 7,0 | APOE 3/? |  |
|  | 56,0  (E) |  |  |  | 10,3 |  | 11,0 | APOE 3/? |  |
|  | 56,0 |  |  |  | 10,3 |  | 11,0 | Not reported |  |
|  | 58,0 |  |  |  | 12,3 |  | 13,0 | Not reported |  |
|  | 58,0 |  |  |  | 12,3 |  | 13,0 | APOE 3/3 |  |
|  | 59,0 |  |  |  | 13,3 |  | 14,0 | Not reported |  |
|  | 68,0  (H) |  |  |  | 22,3 |  | 23,0 | APOE 3/3 |  |
|  | 43,0 |  |  |  | -2,7 |  | -2,0 | Not reported |  |
|  | 52,0 |  |  |  | 6,3 |  | 7,0 | Not reported |  |
|  | 60,0 |  |  |  | 14,3 |  | 15,0 | Not reported |  |
|  | 67,0 |  |  |  | 21,3 |  | 22,0 | APOE 4/4 |  |
|  | 45,0 |  |  |  | -0,7 |  | 0,0 | Not reported |  |
|  | 44,0 |  |  |  | -1,7 |  | -1,0 | APOE 4/? |  |
|  | 40,0 |  |  |  | -5,7 |  | -5,0 | Not reported |  |
|  | 49,0 |  |  |  | 3,3 |  | 4,0 | Not reported |  |
|  | 50,0 |  |  |  | 4,3 |  | 5,0 | APOE 4/? |  |
|  | 56,0 |  |  |  | 10,3 |  | 11,0 | APOE 3/3 |  |
|  | 43,0 |  |  |  | -2,7 |  | -2,0 | Not reported |  |
|  | 45,0 |  |  |  | -0,7 |  | 0,0 | APOE 4/? |  |
|  | 45,0 |  |  |  | -0,7 |  | 0,0 | Not reported |  |
|  | 54,0 |  |  |  | 8,3 |  | 9,0 | APOE 4/4 |  |
|  | 64,0 |  |  |  | 18,3 |  | 19,0 | Not reported |  |
|  | 54,0 |  |  |  | 8,3 |  | 9,0 | APOE 3/? |  |
|  | 55,0 |  |  |  | 9,3 |  | 10,0 | APOE 4/? |  |
|  | 47,0 |  |  |  | 1,3 |  | 2,0 | Not reported |  |
|  | 53,0 |  |  |  | 7,3 |  | 8,0 | APOE 3/4 |  |
|  | 46,0 |  |  |  | 0,3 |  | 1,0 | APOE 3/4 |  |
|  | 45,0  (R) |  |  |  | -0,7 |  | 0,0 | APOE 3/4 |  |
|  | 55,0 |  |  |  | 9,3 |  | 10,0 | Not reported |  |
|  | 75,0 |  |  |  | 29,3 |  | 30,0 | APOE 3/3 |  |
|  | 55,0 |  |  |  | 9,3 |  | 10,0 | APOE 3/3 |  |
|  | 65,0 |  |  |  | 19,3 |  | 20,0 | APOE 3/3 |  |
|  | 72,0 |  |  |  | 26,3 |  | 27,0 | APOE 3/3 |  |
|  | 68,0 |  |  |  | 22,3 |  | 23,0 | APOE 3/3 |  |
|  | 62,0  (HB) |  |  |  | 16,3 |  | 17,0 | Not reported |  |
|  | 70,0 |  |  |  | 24,3 |  | 25,0 | Not reported |  |
|  | 55,0 |  |  |  | 9,3 |  | 10,0 | Not reported |  |
|  | 67,0 |  |  |  | 21,3 |  | 22,0 | Not reported |  |
|  | 53,0 |  |  |  | 7,3 |  | 8,0 | Not reported |  |
|  | 47,0 |  |  |  | 1,3 |  | 2,0 | Not reported |  |
|  | 75,0 |  |  |  | 29,3 |  | 30,0 | APOE 3/4 |  |
|  | 51,0 |  |  |  | 5,3 |  | 6,0 | Not reported |  |
|  | 60,0 |  |  |  | 14,3 |  | 15,0 | APOE 3/3 |  |
|  | 52,0 |  |  |  | 6,3 |  | 7,0 | APOE 3/3 |  |
|  | 46,0 |  |  |  | 0,3 |  | 1,0 | APOE 2/3 |  |
|  | 49,0  (HD) |  |  |  | 3,3 |  | 4,0 | APOE 3/3 |  |
|  | 70,0 |  |  |  | 24,3 |  | 25,0 | Not reported |  |
|  | 59,0 |  |  |  | 13,3 |  | 14,0 | Not reported |  |
|  | 71,0 |  |  |  | 25,3 |  | 26,0 | APOE 3/3 |  |
|  | 68,0 |  |  |  | 22,3 |  | 23,0 | APOE 3/4 |  |
|  | 67,0 |  |  |  | 21,3 |  | 22,0 | APOE 4/4 |  |
|  | 58,0 |  |  |  | 12,3 |  | 13,0 | APOE 4/4 |  |
|  | 71,0 |  |  |  | 25,3 |  | 26,0 | APOE 3/4 |  |
|  | 57,0 |  |  |  | 11,3 |  | 12,0 | Not reported |  |
|  | 67,0 |  |  |  | 21,3 |  | 22,0 | APOE 3/4 |  |
|  | 72,0  (KS) |  |  |  | 26,3 |  | 27,0 | Not reported |  |
|  | 58,0 |  |  |  | 12,3 |  | 13,0 | Not reported |  |
|  | 54,0 |  |  |  | 8,3 |  | 9,0 | APOE 3/4 |  |
|  | 57,0 |  |  |  | 11,3 |  | 12,0 | Not reported |  |
|  | 48,0 |  |  |  | 2,3 |  | 3,0 | APOE 3/4 |  |
|  | 47,0  (W) |  |  |  | 1,3 |  | 2,0 | APOE 3/3 |  |
|  | 76,0 |  |  |  | 30,3 |  | 31,0 | Not reported |  |
|  | 60,0 |  |  |  | 14,3 |  | 15,0 | APOE 3/? |  |
|  | 62,0  (WFL) |  |  |  | 16,3 |  | 17,0 | APOE 3/3 |  |
|  | 51,0 |  |  |  | 5,3 |  | 6,0 | Not reported | Blauwendraat et al.,2015 |
|  | 56,0 |  |  |  | 10,3 |  | 11,0 | Not reported |  |
|  | 60,0 |  |  |  | 14,3 |  | 15,0 | Not reported | Jorge J.Llibre-Guerra et al., 2020;  Carolina Muchnik et al., 2015 |
|  | 55,0 |  |  |  | 9,3 |  | 10,0 | Not reported |  |
|  | 51,0 |  |  |  | 5,3 |  | 6,0 | Not reported |  |
|  | 54,0 |  |  |  | 8,3 |  | 9,0 | Not reported |  |
|  | 50,0 |  |  |  | 4,3 |  | 5,0 | Not reported |  |
|  | 58,0 |  |  |  | 12,3 |  | 13,0 | APOE 3/3 |  |
|  | 53,0 |  |  |  | 7,3 |  | 8,0 | APOE 2/3 |  |
|  | 55,0 |  |  |  | 9,3 |  | 10,0 | APOE 2/3 |  |
|  | 58,0 |  |  |  | 12,3 |  | 13,0 | APOE 2/3 |  |
|  | 50,0 |  |  |  | 4,3 |  | 5,0 | Not reported |  |
|  | 52,0 |  |  |  | 6,3 |  | 7,0 | APOE 3/3 |  |
|  | 50,0  (AR2) |  |  |  | 4,3 |  | 5,0 | APOE 2/3 |  |
|  | 50,0 |  |  |  | 4,3 |  | 5,0 | Not reported |  |
|  | 50,0  (AR3) |  |  |  | 4,3 |  | 5,0 | APOE 4/4 |  |
| N141S | 52,0 | 52,0 | / | 49,8 | 2,2 | 51,9 | 0,1 | APOE 3/4 | Mao et al., 2021 |
| N141Y | 43,0 | 46,0 | 4,2 | 45,9 | -2,9 | 45,5 | -2,5 | APOE 3/3 | Niu et al., 2014 |
|  | 49,0 |  |  |  | 3,1 |  | 3,5 | APOE 3/3 |  |
| I149T | 63,0 | 63,0 | / | 62,4 | 0,6 | 61,8 | 1,2 | Not reported | Perrone et al., 2020 |
| K161R | 65,0 | 65,0 | / | / | / | / | / | Not reported | Wallon et al., 2012 |
| H169N | 68,0 | 62,5 | 5,0 | / | / | / | / | APOE 3/4 | Shi et al., 2015 |
|  | 62,0 |  |  |  |  |  |  | Not reported | Shi et al., 2015 |
|  | 64,0 |  |  |  |  |  |  | APOE 3/3 | Xu et al., 2018 |
|  | 56,0 |  |  |  |  |  |  | APOE 3/3 | Giau et al., 2018 |
| S175C | 60,0 | 62,0 | 2,6 | 67,3 | -7,3 | 64,2 | -4,2 | APOE 3/4 | Piscopo et al., 2010 |
|  | 61,0 |  |  |  | -6,3 |  | -3,2 | APOE 3/4 |  |
|  | 65,0 |  |  |  | -2,3 |  | 0,8 | APOE 3/4 |  |
| S175F | 52,0 | 53,0 | 4,6 | 52,1 | -0,1 | 52,8 | -0,8 | APOE 3/4 | Guven et al., 2021 |
|  | 49,0 |  |  |  | -3,1 |  | -3,8 | APOE 3/3 |  |
|  | 58,0 |  |  |  | 5,9 |  | 5,2 | Not reported |  |
| G212V | 65,0 | 61,5 | 2,4 | 57,0 | 8,0 | 58,3 | 6,7 | APOE 3/3 | Marín-Muñoz et al., 2016 |
|  | 61,0 |  |  |  | 4,0 |  | 2,7 | Not reported |  |
|  | 60,0 |  |  |  | 3,0 |  | 1,7 | Not reported |  |
|  | 60,0 |  |  |  | 3,0 |  | 1,7 | Not reported |  |
| I235F | 57,0 | 57,0 | / | / | / | / | / | Not reported | Lee et al., 2014 |
| L238F | 74,0 | 59,8 | 10,4 | ND | / | ND | / | APOE 3/3 | Frigerio et al., 2015 |
|  | 49,0 |  |  |  |  |  |  | Not reported | Hsu et al., 2018 |
|  | 57,0 |  |  |  |  |  |  | Not reported | Hsu et al., 2018 |
|  | 59,0 |  |  |  |  |  |  | Not reported | N.Ryan, personal communication |
| M239I | 44,0 | 50,1 | 7,2 | 51,9 | -7,9 | 51,2 | -7,2 | APOE 3/3 | Finckh et al., 2000 |
|  | 50,0 |  |  |  | -1,9 |  | -1,2 | APOE 3/3 |  |
|  | 58,0 |  |  |  | 6,1 |  | 6,8 | APOE 3/3 |  |
|  | 56,0 |  |  |  | 4,1 |  | 4,8 | Not reported |  |
|  | 50,0 |  |  |  | -1,9 |  | -1,2 | APOE 3/3 | Testi et al., 2012 |
|  | 45,0 |  |  |  | -6,9 |  | -6,2 | APOE 3/3 |  |
|  | 50,0 |  |  |  | -1,9 |  | -1,2 | APOE 3/3 |  |
|  | 50,0 |  |  |  | -1,9 |  | -1,2 | APOE 3/3 | Tremolizzo et al., 2014 |
|  | 58,0 |  |  |  | 6,1 |  | 6,8 | Not reported |  |
|  | 55,0 |  |  |  | 3,1 |  | 3,8 | Not reported |  |
|  | 55,0 |  |  |  | 3,1 |  | 3,8 | Not reported |  |
|  | 30,0 |  |  |  | -21,9 |  | -21,2 | Not reported |  |
|  | 49,0 |  |  |  | -2,9 |  | -2,2 | Not reported | Llibre-Guerra et al., 2021 |
|  | 51,0 |  |  |  | -0,9 |  | -0,2 | Not reported |  |
|  | 50,0 |  |  |  | -1,9 |  | -1,2 | Not reported |  |
|  | 50,0 |  |  |  | -1,9 |  | -1,2 | APOE 3/4 | Jiao et al., 2021 |
| M239V | 83,0 | 57,2 | 8,9 | 49,6 | 33,4 | 47,7 | 35,3 | Not reported | Marcon et al., 2004 |
|  | 70,0 |  |  |  | 20,4 |  | 22,3 | Not reported |  |
|  | 49,0 |  |  |  | -0,6 |  | 1,3 | Not reported |  |
|  | 60,0 |  |  |  | 10,4 |  | 12,3 | Not reported |  |
|  | 60,0 |  |  |  | 10,4 |  | 12,3 | Not reported |  |
|  | 56,0 |  |  |  | 6,4 |  | 8,3 | Not reported |  |
|  | 60,0 |  |  |  | 10,4 |  | 12,3 | Not reported |  |
|  | 62,0 |  |  |  | 12,4 |  | 14,3 | Not reported |  |
|  | 60,0 |  |  |  | 10,4 |  | 12,3 | Not reported |  |
|  | 73,0 |  |  |  | 23,4 |  | 25,3 | Not reported |  |
|  | 45,0 |  |  |  | -4,6 |  | -2,7 | Not reported |  |
|  | 48,0 |  |  |  | -1,6 |  | 0,3 | Not reported |  |
|  | 52,0 |  |  |  | 2,4 |  | 4,3 | Not reported |  |
|  | 58,0 |  |  |  | 8,4 |  | 10,3 | Not reported |  |
|  | 66,0  (Flo10) |  |  |  | 16,4 |  | 18,3 | Not reported |  |
|  | 47,0 |  |  |  | -2,6 |  | -0,7 | APOE 3/4 | Wallon et al., 2012 |
|  | 55,0  (Alz400) |  |  |  | 5,4 |  | 7,3 | Not reported |  |
|  | 53,0 |  |  |  | 3,4 |  | 5,3 | APOE 3/4 |  |
|  | 62,0  (Tou035) |  |  |  | 12,4 |  | 14,3 | Not reported |  |
|  | 48,0 |  |  |  | -1,6 |  | 0,3 | APOE 3/4 |  |
|  | 67,0  (Alz434) |  |  |  | 17,4 |  | 19,3 | Not reported |  |
|  | 49,0 |  |  |  | -0,6 |  | 1,3 | APOE 3/4 |  |
|  | 57,0  (Rou360) |  |  |  | 7,4 |  | 9,3 | Not reported |  |
|  | 47,0 |  |  |  | -2,6 |  | -0,7 | APOE 3/4 |  |
|  | 60,0  (Ext062) |  |  |  | 10,4 |  | 12,3 | Not reported |  |
|  | 53,0 |  |  |  | 3,4 |  | 5,3 | APOE 2/4 | Nicholas et al., 2015 |
|  | 49,0 |  |  |  | -0,6 |  | 1,3 | Not reported | Li et al., 2021 |
|  | 53,0 |  |  |  | 3,4 |  | 5,3 | APOE 2/3 | Jiao et al., 2021 |
| M239T | 59,0 | 52,0 | 6,2 | 59,6 | -0,6 | 56,0 | 3,0 | APOE 3/4 | Li et al., 2021 |
|  | 50,0 |  |  |  | -9,6 |  | -6,0 | APOE 3/4 | Jiao et al., 2021 |
|  | 47,0 |  |  |  | -12,6 |  | -9,0 | APOE 3/3 | Mao et al., 2021 |
| R284G | 57,0 | 57,5 | 0,7 | 59,5 | -2,5 | 65,2 | -8,2 | APOE 3/4 | Lanoiselée et al., 2017 |
|  | 58,0 |  |  |  | -1,5 |  | -7,2 | Not reported | Hsu et al., 2018 |
| M298T | 56,0 | 57,2 | 1,3 | / | / | / | / | APOE 3/3 | Wang et al., 2019 |
|  | 58,0 |  |  |  |  |  |  | APOE 3/3 | Jia et al., 2020 |
|  | 59,0 |  |  |  |  |  |  | APOE 3/3 |  |
|  | 57,0 |  |  |  |  |  |  | APOE 3/3 |  |
|  | 56,0 |  |  |  |  |  |  | APOE 3/4 | Mao et al., 2021 |
| A379D | 55,0 | 55,0 | / | / | / | / | / | APOE 3/3 | Wang et al., 2019 |
| P69A | 74,0 | 74,0 | / | / | / | / | / | Not reported | Dobricic et al., 2012 |
| R71W | 75,0 | 63,4 | 5,1 | / | / | / | / | Not reported | Sleegers et al., 2004 |
|  | 64,0 |  |  |  |  |  |  | Not reported | Lohmann et al., 2012 |
|  | 63,0 |  |  |  |  |  |  | Not reported | Wallon et al., 2012 |
|  | 64,0 |  |  |  |  |  |  | Not reported |  |
|  | 55,0 |  |  |  |  |  |  | Not reported |  |
|  | 65,0 |  |  |  |  |  |  | Not reported | Schulte et al., 2012 |
|  | 60,0 |  |  |  |  |  |  | APOE 3/3 | Nicolas et al., 2015 |
|  | 65,0 |  |  |  |  |  |  | APOE 3/4 |  |
|  | 60,0 |  |  |  |  |  |  | Not reported | Coppola et al., 2021 |
| V214L | 69,0 | 57,3 | 7,0 | / | / | / | / | Not reported | Youn et al., 2014 |
|  | 54,0 |  |  |  |  |  |  | Not reported | An et al., 2016 |
|  | 64,0 |  |  |  |  |  |  | Not reported | Shi et al., 2015 |
|  | 63,0 |  |  |  |  |  |  | APOE 3/3 |  |
|  | 52,0 |  |  |  |  |  |  | Not reported | Xu et al., 2018 |
|  | 54,0 |  |  |  |  |  |  | APOE 3/4 |  |
|  | 53,0 |  |  |  |  |  |  | Not reported | Jia et al., 2020 |
|  | 42,0 |  |  |  |  |  |  | Not reported |  |
|  | 58,0 |  |  |  |  |  |  | Not reported |  |
|  | 60,0 |  |  |  |  |  |  | APOE 3/4 |  |
|  | 61,0 |  |  |  |  |  |  | APOE 3/4 |  |
| P334A | Not report | / | / | / | / | / | / | Not reported | Lee et al., 2014 |
| T421M | 55 | 55 | / | / | / | / | / | APOE 4/4 | Yagi et al., 2014 |

| **Supplementary Table S2** | | | | | | | | | |
| --- | --- | --- | --- | --- | --- | --- | --- | --- | --- |
| **Mutation in  APP TMD** | **AAO cases** | **Mean  AAO** | **SD** | **AAO predicted  GSEC processivity** | **AAO- AAO predicted  (GSEC processivity)** | **AAO predicted  product line ratio** | **AAO- AAO predicted  (Product line)** | **APOE** | **References** |
| **L705V** | 63,0 | 63,4 | 8,6 | / | / | / | **/** | Not reported | Obici et al., 2005 |
|  | 50,0 |  |  |  |  |  |  | Not reported |  |
|  | 72,0 |  |  |  |  |  |  | Not reported |  |
|  | 70,0 |  |  |  |  |  |  | Not reported |  |
|  | 62,0 |  |  |  |  |  |  | Not reported | Kozberg et al., 2020 |
| **A713T** | 59,0 | 61,3 | 8,8 | 71,9 | -12,9 | 72,2 | -13,2 | Not reported | Carter et al., 1992 |
|  | 52,0 |  |  |  | -19,9 |  | -20,2 | Not reported |  |
|  | 57,0 |  |  |  | -14,9 |  | -15,2 | Not reported |  |
|  | 56,0  (Italian) |  |  |  | -15,9 |  | -16,2 | Not reported | Rossi et al., 2004 |
|  | 49,0 |  |  |  | -22,9 |  | -23,2 | Not reported | Armstrong et al., 2004 |
|  | 62,0 |  |  |  | -9,9 |  | -10,2 | APOE 3/3 |  |
|  | 73,0 |  |  |  | 1,1 |  | 0,8 | APOE 3/3 |  |
|  | 76,0 |  |  |  | 4,1 |  | 3,8 | APOE 2/3 |  |
|  | 70,0 |  |  |  | -1,9 |  | -2,2 | APOE 3/3 | Conidi et al., 2015 |
|  | 70,0  (PEC) |  |  |  | -1,9 |  | -2,2 | APOE 3/3 |  |
|  | 62,0 |  |  |  | -9,9 |  | -10,2 | Not reported | Barber et al., 2016 |
|  | 61,0 |  |  |  | -10,9 |  | -11,2 | APOE 3/4 |  |
|  | 50,0 |  |  |  | -21,9 |  | -22,2 | Not reported | Lombardi et al., 2017 |
| **T714A** | 44,0 | 53,0 | 11,2 | 50,2 | -6,2 | 47,9 | -3,9 | Not reported | Zekanowski et al., 2003 |
|  | 47,0 |  |  |  | -3,2 |  | -0,9 | Not reported | Lindquist et al., 2008 |
|  | 52,0 |  |  |  | 1,8 |  | 4,1 | Not reported | Pasalar et al., 2002 |
|  | 58,0 |  |  |  | 7,8 |  | 10,1 | Not reported |  |
|  | 69,0 |  |  |  | 18,8 |  | 21,1 | Not reported |  |
|  | 52,0 |  |  |  | 1,8 |  | 4,1 | Not reported |  |
|  | 54,0 |  |  |  | 3,8 |  | 6,1 | Not reported |  |
|  | 52,0 |  |  |  | 1,8 |  | 4,1 | Not reported |  |
|  | 52,0 |  |  |  | 1,8 |  | 4,1 | Not reported |  |
|  | 50,0  (Iranian) |  |  |  | -0,2 |  | 2,1 | Not reported |  |
| **T714I** | 32,0 | 37,4 | 3,9 | 39,0 | -7,0 | 37,3 | -5,3 | APOE 3/3 | Kumar-Singh et al., 2000 |
|  | 32,0 |  |  |  | -7,0 |  | -5,3 | APOE 3/4 |  |
|  | 38,0 |  |  |  | -1,0 |  | 0,7 | APOE 2/3 |  |
|  | 41,0 |  |  |  | 2,0 |  | 3,7 | Not reported |  |
|  | 38,0 |  |  |  | -1,0 |  | 0,7 | Not reported | Edwards-Lee et al., 2005 |
|  | 39,0 |  |  |  | 0,0 |  | 1,7 | Not reported |  |
|  | 42,0 |  |  |  | 3,0 |  | 4,7 | Not reported |  |
| **V715A** | 50,0 | 48,7 | 5,9 | 47,3 | 2,7 | 45,9 | 4,1 | Not reported | Cruts et al., 2003 |
|  | 55,0 |  |  |  | 7,7 |  | 9,1 | Not reported |  |
|  | 55,0 |  |  |  | 7,7 |  | 9,1 | Not reported |  |
|  | 48,0 |  |  |  | 0,7 |  | 2,1 | Not reported |  |
|  | 42,0 |  |  |  | -5,3 |  | -3,9 | Not reported | Zekanowski et al., 2003 |
|  | 42,0 |  |  |  | -5,3 |  | -3,9 | Not reported | Wallon et al., 2012 |
| **V715M** | 44,0 | 49,3 | 8,5 | 39,7 | 4,3 | 40,1 | 3,9 | APOE 3/3 | Ancolio et al., 1999 |
|  | 60,0 |  |  |  | 20,3 |  | 19,9 | Not reported |  |
|  | 52,0 |  |  |  | 12,3 |  | 11,9 | APOE 2/3 |  |
|  | 41,0 |  |  |  | 1,3 |  | 0,9 | Not reported | Park et al., 2008 |
| **I716F** | 31,0 | 33,7 | 6,7 | 35,3 | -4,3 | 34,4 | -3,4 | APOE 3/3 | Guerreiro et al., 2010 |
|  | 47,0 |  |  |  | 11,7 |  | 12,6 | Not reported | Sieczkowski et al., 2015 |
|  | 34,0 |  |  |  | -1,3 |  | -0,4 | Not reported |  |
|  | 30,0 |  |  |  | -5,3 |  | -4,4 | Not reported |  |
|  | 30,0 |  |  |  | -5,3 |  | -4,4 | Not reported |  |
|  | 30,0 |  |  |  | -5,3 |  | -4,4 | Not reported |  |
| **I716M** | 64,0 | 64,0 | / | 56,8 | 7,2 | 59,4 | 4,6 | Not reported | Blauwendraat et al., 2016 |
| **I716T** | 36,0 | 36,0 | / | 39,6 | -3,6 | 41,1 | -5,1 | Not reported | Terreni et al., 2002 |
| **I716V** | 53,0 | 55,7 | 2,1 | 66,6 | -13,6 | 62,3 | -9,3 | Not reported | Eckman et al., 1997 |
|  | 56,0 |  |  |  | -10,6 |  | -6,3 | Not reported | N.Ryan, personal communication |
|  | 58,0 |  |  |  | -8,6 |  | -4,3 | Not reported |  |
| **V717F** | 38,0 | 45,0 | 4,7 | 48,3 | -10,3 | 48,4 | -10,4 | Not reported | Finckh et al., 2005 |
|  | 40,0 |  |  |  | -8,3 |  | -8,4 | Not reported |  |
|  | 37,0 |  |  |  | -11,3 |  | -11,4 | Not reported |  |
|  | 41,0 |  |  |  | -7,3 |  | -7,4 | Not reported | Murrell et al., 1991 |
|  | 42,0 |  |  |  | -6,3 |  | -6,4 | Not reported |  |
|  | 45,0 |  |  |  | -3,3 |  | -3,4 | Not reported |  |
|  | 44,0 |  |  |  | -4,3 |  | -4,4 | Not reported |  |
|  | 40,0 |  |  |  | -8,3 |  | -8,4 | Not reported | Zádori et al., 2017 |
|  | 40,0 |  |  |  | -8,3 |  | -8,4 | Not reported |  |
|  | 50,0 |  |  |  | 1,7 |  | 1,6 | Not reported |  |
|  | 51,0 |  |  |  | 2,7 |  | 2,6 | Not reported |  |
|  | 52,0  (Japanese) |  |  |  | 3,7 |  | 3,6 | Not reported |  |
|  | 50,0 |  |  |  | 1,7 |  | 1,6 | Not reported | Abe et al., 2012 |
|  | 50,0 |  |  |  | 1,7 |  | 1,6 | Not reported |  |
|  | 42,0 |  |  |  | -6,3 |  | -6,4 | Not reported |  |
|  | 52,0 |  |  |  | 3,7 |  | 3,6 | Not reported |  |
|  | 47,0 |  |  |  | -1,3 |  | -1,4 | Not reported |  |
|  | 46,0 |  |  |  | -2,3 |  | -2,4 | Not reported |  |
|  | 45,0 |  |  |  | -3,3 |  | -3,4 | APOE 3/3 |  |
|  | 45,0 |  |  |  | -3,3 |  | -3,4 | APOE 3/3 |  |
|  | 47,0 |  |  |  | -1,3 |  | -1,4 | APOE 3/3 |  |
| **V717G** | 61,0 | 52,5 | 7,2 | 54,7 | 6,3 | 55,9 | 5,1 | APOE 3/3 | Chartier-Harlin et al., 1991 |
|  | 40,0 |  |  |  | -14,7 |  | -15,9 | Not reported | N.Ryan, personal communication |
|  | 46,0 |  |  |  | -8,7 |  | -9,9 | Not reported |  |
|  | 44,0 |  |  |  | -10,7 |  | -11,9 | Not reported |  |
|  | 48,0 |  |  |  | -6,7 |  | -7,9 | Not reported |  |
|  | 50,0 |  |  |  | -4,7 |  | -5,9 | Not reported |  |
|  | 55,0 |  |  |  | 0,3 |  | -0,9 | Not reported |  |
|  | 61,0 |  |  |  | 6,3 |  | 5,1 | Not reported |  |
|  | 56,0 |  |  |  | 1,3 |  | 0,1 | Not reported |  |
|  | 59,0 |  |  |  | 4,3 |  | 3,1 | Not reported |  |
|  | 53,0 |  |  |  | -1,7 |  | -2,9 | Not reported |  |
|  | 45,0 |  |  |  | -9,7 |  | -10,9 | Not reported |  |
|  | 50,0 |  |  |  | -4,7 |  | -5,9 | Not reported |  |
|  | 51,0 |  |  |  | -3,7 |  | -4,9 | Not reported |  |
|  | 58,0 |  |  |  | 3,3 |  | 2,1 | Not reported |  |
|  | 67,0  (F19) |  |  |  | 12,3 |  | 11,1 | Not reported |  |
|  | 48,0 |  |  |  | -6,7 |  | -7,9 | Not reported | Küçükali et al., 2022 |
| **V717I** | 58,0 | 53,9 | 4,8 | 59,1 | -1,1 | 58,6 | -0,6 | Not reported | Mullan et al., 1992 |
|  | 55,0  (G) |  |  |  | -4,1 |  | -3,6 | Not reported |  |
|  | 45,0 |  |  |  | -14,1 |  | -13,6 | Not reported |  |
|  | 52,0 |  |  |  | -7,1 |  | -6,6 | Not reported |  |
|  | 52,0  (Japanese) |  |  |  | -7,1 |  | -6,6 | Not reported |  |
|  | 57,0 |  |  |  | -2,1 |  | -1,6 | Not reported | Mullan et al., 1993 |
|  | 52,0 |  |  |  | -7,1 |  | -6,6 | Not reported |  |
|  | 57,0 |  |  |  | -2,1 |  | -1,6 | Not reported |  |
|  | 52,0 |  |  |  | -7,1 |  | -6,6 | Not reported |  |
|  | 52,0 |  |  |  | -7,1 |  | -6,6 | Not reported |  |
|  | 59,0 |  |  |  | -0,1 |  | 0,4 | Not reported |  |
|  | 58,0 |  |  |  | -1,1 |  | -0,6 | Not reported |  |
|  | 58,0 |  |  |  | -1,1 |  | -0,6 | Not reported |  |
|  | 53,0 |  |  |  | -6,1 |  | -5,6 | Not reported |  |
|  | 51,0  (F23) |  |  |  | -8,1 |  | -7,6 | Not reported |  |
|  | 55,0 |  |  |  | -4,1 |  | -3,6 | APOE 2/3 |  |
|  | 50,0 |  |  |  | -9,1 |  | -8,6 | Not reported |  |
|  | 48,0  (Nigata1) |  |  |  | -11,1 |  | -10,6 | Not reported |  |
|  | 55,0 |  |  |  | -4,1 |  | -3,6 | Not reported |  |
|  | 59,0 |  |  |  | -0,1 |  | 0,4 | Not reported |  |
|  | 52,0 |  |  |  | -7,1 |  | -6,6 | Not reported |  |
|  | 51,0  (Nigata2) |  |  |  | -8,1 |  | -7,6 | Not reported |  |
|  | 59,0 |  |  |  | -0,1 |  | 0,4 | Not reported |  |
|  | 41,0 |  |  |  | -18,1 |  | -17,6 | Not reported |  |
|  | 49,0 |  |  |  | -10,1 |  | -9,6 | Not reported |  |
|  | 48,0 |  |  |  | -11,1 |  | -10,6 | Not reported |  |
|  | 50,0  (372) |  |  |  | -9,1 |  | -8,6 | Not reported |  |
|  | 52,0 |  |  |  | -7,1 |  | -6,6 | Not reported | Zhang et al., 2016 |
|  | 45,0 |  |  |  | -14,1 |  | -13,6 | Not reported |  |
|  | 55,0 |  |  |  | -4,1 |  | -3,6 | APOE 3/3 |  |
|  | 44,0 |  |  |  | -15,1 |  | -14,6 | APOE 4/4 |  |
|  | 46,0  (Chinese1) |  |  |  | -13,1 |  | -12,6 | APOE 3/3 |  |
|  | 58,0 |  |  |  | -1,1 |  | -0,6 | Not reported |  |
|  | 60,0 |  |  |  | 0,9 |  | 1,4 | Not reported |  |
|  | 57,0 |  |  |  | -2,1 |  | -1,6 | Not reported |  |
|  | 55,0 |  |  |  | -4,1 |  | -3,6 | APOE 3/3 |  |
|  | 51,0  (Chinese2) |  |  |  | -8,1 |  | -7,6 | APOE 3/4 |  |
|  | 60,0 |  |  |  | 0,9 |  | 1,4 | Not reported |  |
|  | 61,0 |  |  |  | 1,9 |  | 2,4 | Not reported |  |
|  | 60,0 |  |  |  | 0,9 |  | 1,4 | Not reported |  |
|  | 62,0 |  |  |  | 2,9 |  | 3,4 | APOE 3/3 |  |
|  | 58,0 |  |  |  | -1,1 |  | -0,6 | APOE 3/3 |  |
|  | 55,0 |  |  |  | -4,1 |  | -3,6 | APOE 3/3 |  |
|  | 59,0 |  |  |  | -0,1 |  | 0,4 | APOE 3/3 |  |
|  | 59,0 |  |  |  | -0,1 |  | 0,4 | APOE 3/3 |  |
|  | 54,0 |  |  |  | -5,1 |  | -4,6 | APOE 4/4 |  |
|  | 54,0  (Chinese3) |  |  |  | -5,1 |  | -4,6 | APOE 3/3 |  |
|  | 52,0 |  |  |  | -7,1 |  | -6,6 | Not reported |  |
|  | 59,0 |  |  |  | -0,1 |  | 0,4 | Not reported |  |
|  | 57,0 |  |  |  | -2,1 |  | -1,6 | Not reported |  |
|  | 58,0 |  |  |  | -1,1 |  | -0,6 | Not reported |  |
|  | 56,0 |  |  |  | -3,1 |  | -2,6 | Not reported |  |
|  | 54,0 |  |  |  | -5,1 |  | -4,6 | APOE 3/4 |  |
|  | 60,0 |  |  |  | 0,9 |  | 1,4 | Not reported |  |
|  | 59,0 |  |  |  | -0,1 |  | 0,4 | APOE 3/3 |  |
|  | 55,0 |  |  |  | -4,1 |  | -3,6 | APOE 3/3 |  |
|  | 47,0  (Chinese4) |  |  |  | -12,1 |  | -11,6 | APOE 4/4 |  |
|  | 50,0 |  |  |  | -9,1 |  | -8,6 | Not reported |  |
|  | 52,0 |  |  |  | -7,1 |  | -6,6 | APOE 3/3 |  |
|  | 47,0 |  |  |  | -12,1 |  | -11,6 | APOE 3/4 |  |
|  | 50,0  (Chinese5) |  |  |  | -9,1 |  | -8,6 | APOE 3/4 |  |
| **V717L** | 38,0 | 46,9 | 6,0 | 51,5 | -13,5 | 49,7 | -11,7 | APOE 3/3 | Godbolt et al., 2006 |
|  | 35,0 |  |  |  | -16,5 |  | -14,7 | APOE 3/3 |  |
|  | 36,0 |  |  |  | -15,5 |  | -13,7 | APOE 3/3 |  |
|  | 48,0 |  |  |  | -3,5 |  | -1,7 | APOE 3/3 |  |
|  | 48,0 |  |  |  | -3,5 |  | -1,7 | APOE 3/3 |  |
|  | 48,0 |  |  |  | -3,5 |  | -1,7 | APOE 3/3 |  |
|  | 57,0 |  |  |  | 5,5 |  | 7,3 | APOE 3/3 |  |
|  | 48,0 |  |  |  | -3,5 |  | -1,7 | APOE 3/3 |  |
|  | 51,0  (171) |  |  |  | -0,5 |  | 1,3 | APOE 3/3 |  |
|  | 50,0 |  |  |  | -1,5 |  | 0,3 | APOE 3/3 | Abe et al., 2012 |
|  | 50,0 |  |  |  | -1,5 |  | 0,3 | APOE 3/3 |  |
|  | 42,0 |  |  |  | -9,5 |  | -7,7 | APOE 3/3 |  |
|  | 52,0 |  |  |  | 0,5 |  | 2,3 | APOE 3/3 |  |
|  | 47,0 |  |  |  | -4,5 |  | -2,7 | APOE 3/3 |  |
|  | 46,0 |  |  |  | -5,5 |  | -3,7 | APOE 3/3 |  |
|  | 45,0 |  |  |  | -6,5 |  | -4,7 | APOE 3/3 |  |
|  | 45,0 |  |  |  | -6,5 |  | -4,7 | APOE 3/3 |  |
|  | 47,0  (Japanese) |  |  |  | -4,5 |  | -2,7 | APOE 3/3 |  |
|  | 50,0 |  |  |  | -1,5 |  | 0,3 | Not reported | Finckh et al., 2005 |
|  | 43,0 |  |  |  | -8,5 |  | -6,7 | Not reported | Finckh et al., 2005 |
|  | 59,0 |  |  |  | 7,5 |  | 9,3 | APOE 3/3 | Sassi et al., 2014 |
|  | 58,0 |  |  |  | 6,5 |  | 8,3 | APOE 3/3 | Hooli et al., 2012 |
|  | 45,0 |  |  |  | -6,5 |  | -4,7 | APOE 3/4 |  |
|  | 46,0 |  |  |  | -5,5 |  | -3,7 | APOE 3/4 |  |
|  | 50,0  (VII) |  |  |  | -1,5 |  | 0,3 | APOE 3/4 |  |
|  | 35,0 |  |  |  | -16,5 |  | -14,7 | Not reported | Ghetti et al., 2008 |
| **T719N** | 46,0 | 45,5 | 0,7 | 42,7 | 3,3 | 45,4 | 0,6 | Not reported | Scahill et al., 2013 |
|  | 45,0 |  |  |  | 2,3 |  | -0,4 | Not reported | Hsu et al., 2018 |
| **T719P** | 43,0 | 43,0 | / | 38,7 | 4,3 | 39,1 | 3,9 | APOE 3/3 | Ghidoni et al., 2009 |
| **M722K** | 38,0 | 49,2 | 6,5 | 43,7 | -5,7 | 44,5 | -6,5 | APOE 3/4 | Wang et al., 2015 |
|  | 52,0 |  |  |  | 8,3 |  | 7,5 | Not reported |  |
|  | 51,0 |  |  |  | 7,3 |  | 6,5 | APOE 3/3 |  |
|  | 49,0 |  |  |  | 5,3 |  | 4,5 | APOE 3/4 |  |
|  | 56,0 |  |  |  | 12,3 |  | 11,5 | Not reported |  |
| **L723P** | 45,0 | 47,0 | 7,8 | 44,6 | 0,4 | 46,7 | -1,7 | APOE 3/4 | Dobricic et al., 2012 |
|  | 40,0 |  |  |  | -4,6 |  | -6,7 | Not reported | Kwok et al., 2000 |
|  | 56,0 |  |  |  | 11,4 |  | 9,3 | Not reported |  |
| **K724N** | 55,0 | 53,5 | 1,5 | 51,1 | 3,9 | 51,4 | 3,6 | APOE 3/4 | Theuns et al., 2006 |
|  | 52,0 |  |  |  | 0,9 |  | 0,6 | Not reported |  |

| **Supplementary Table S3** | | | | | | | |
| --- | --- | --- | --- | --- | --- | --- | --- |
| **Mutation in  PSEN1** | **AAO cases** | **Mean  AAO** | **SD** | **AAO predicted  GSEC processivity** | **AAO- AAO predicted  (GSEC processivity)** | **APOE** | **References** |
| **F388S** | 24,0 | 25,5 | 1,5 | 23,1 | 0,9 | Not reported | Ringman et al., 2023 |
|  | 27,0 |  |  |  | 3,9 | Not reported |  |
| **I180F** | 56,0 | 59,0 | 3,0 | 55,2 | 0,8 | APOE 3/4 | Robbie et al., 2024 |
|  | 62,0 |  |  |  | 6,8 | APOE 3/4 |  |
| **G183V** | 30,0 | 51,2 | 10,9 | 58,1 | -28,1 | Not reported | Dermaut et al., 2004 |
|  | 52,0 |  |  |  | -6,1 | APOE 3/3 | Eryilmaz et al., 2021 |
|  | 60,0 |  |  |  | 1,9 | APOE 3/3 |  |
|  | 58,0 |  |  |  | -0,1 | APOE 3/3 |  |
|  | 56,0 |  |  |  | -2,1 | APOE 3/3 |  |
| **V142I** | 54,0 | 53,3 | 0,9 | 48,4 | 5,6 | Not reported | Koriath et al., 2018 |
|  | 54,0 |  |  |  | 5,6 | APOE 3/3 |  |
|  | 52,0 |  |  |  | 3,6 | APOE 3/3 |  |
| **G266C** | 35,0 |  |  | 56,2 | -21,2 | Not reported | Matsubara-Tsutsui et al., 2002 |
|  | 38,0 |  |  |  | -18,2 | Not reported |  |
|  | 40,0 | 43,5 | 6,1 |  | -16,2 | Not reported |  |
|  | 49,0 |  |  |  | -7,2 | Not reported |  |
|  | 48,0 |  |  |  | -8,2 | Not reported |  |
|  | 51,0 |  |  |  | -5,2 | Not reported |  |
| **L282P** | 41,0 | 41,0 | 0,0 | 47,8 | -6,8 | APOE 3/4 | Kim et al., 2020 |
| **V393F** | 61,0 | 61,0 | 0,0 | 52,8 | 8,2 | APOE 2/3 | Koriath et al., 2018 |
| **T291P** | 43,0 | 39,0 | 5,0 | 36,6 | 6,4 | APOE 3/3 | Dumanchin et al., 2006  Chelban et al., 2021 |
|  | 35,0 |  |  |  | -1,6 | APOE 3/3 |  |
| **Y154N** | 40,0 | 40,0 | 0,0 | 42,7 | -2,7 | APOE 3/3 | Hattori et al., 2004 |
| **L113P** | 38,0 | 42,4 | 4,5 | 44,5 | -6,5 | Not reported |  |
|  | 39,0 |  |  |  | -5,5 | Not reported |  |
|  | 40,0 |  |  |  | -4,5 | Not reported | Raux et al., 2000 |
|  | 45,0 |  |  |  | 0,5 | Not reported |  |
|  | 50,0 |  |  |  | 5,5 | Not reported |  |
| **P88L** | 41,0 | 44,3 | 5,0 | 17,6 | 23,4 | Not reported | Liu et al., 2017 |
|  | 39,0 |  |  |  | 21,4 | Not reported |  |
|  | 45,0 |  |  |  | 27,4 | Not reported | Vazquez-Costa et al., 2021 |
|  | 52,0 |  |  |  | 34,4 | APOE3/3 | Islam et al., 2022 |
| **R278I** | 48 | 49,3 | 1,24 | 18,1 | 29,9 | APOE 2/3 |  |
|  | 51 |  |  |  | 32,9 | APOE 3/4 | Godbolt et al., 2004 |
|  | 49 |  |  |  | 30,9 | Not reported |  |
| **C410Y** | 61,0 | 58,6 |  | 27,4 | 33,6 | Not reported |  |
|  | 61,0 |  | 5,5 |  | 33,6 | Not reported |  |
|  | 57,0 |  |  |  | 29,6 | Not reported |  |
|  | 59,0 |  |  |  | 31,6 | Not reported |  |
|  | 62,0 |  |  |  | 34,6 | Not reported |  |
|  | 64,0 |  |  |  | 36,6 | Not reported |  |
|  | 62,0 |  |  |  | 34,6 | Not reported | Sherrington et al., 1995 |
|  | 60,0 |  |  |  | 32,6 | Not reported |  |
|  | 62,0 |  |  |  | 34,6 | Not reported |  |
|  | 61,0 |  |  |  | 33,6 | Not reported |  |
|  | 55,0 |  |  |  | 27,6 | Not reported |  |
|  | 53.0 |  |  |  | 25,6 | Not reported | Goudsmit et al., 1981 |
|  | 52,0 |  |  |  | 24,6 | Not reported |  |
|  | 70,0 |  |  |  | 42,6 | Not reported |  |
|  | 68,0 |  |  |  | 40,6 | Not reported |  |
|  | 58,0 |  |  |  | 30,6 | Not reported | Campion et al., 1995, |
|  | 56,0 |  |  |  | 28,6 | Not reported |  |
|  | 55,0 |  |  |  | 27,6 | Not reported |  |
|  | 60,0 |  |  |  | 21,6 |  |  |
|  | 55,0 |  |  |  | 20,6 |  |  |
|  | 49,0 |  |  |  | 21,6 | Not reported |  |
|  | 48,0 |  |  |  | 20,6 | Not reported |  |
| **P433S** | 43 | 38,5 | 4,5 | 23,1 | 19,9 | Not reported | Koriath et al., 2018 |
|  | 34 |  |  |  | 10,9 | Not reported |  |
| **L435F** | 59 | 56,5 | 2,5 | 19,6 | 39,4 | Not reported | Heilig et al., 2010 |
|  | 54 |  |  |  | 34,4 | Not reported |  |

**References Table S1 (PSEN2)**

1. Finckh U, Kuschel C, Anagnostouli M, Patsouris E, Pantes G V., Gatzonis S, et al. Novel mutations and repeated findings of mutations in familial Alzheimer disease. Neurogenetics. 2005 May;6(2):85–9.

2. Finckh U, Mu T, Mann U, Eggers C, Marksteiner J, Meins W, et al. High Prevalence of Pathogenic Mutations in Patients with Early-Onset Dementia Detected by Sequence Analyses of Four Different Genes. The American Journal of Human Genetics. 2000;5(1):110–7.

3. Piscopo P, Marcon G, Piras MR, Crestini A, Malvezzi L, Deiana CE, et al. A novel PSEN2 mutation associated with a peculiar phenotype.Neurology. 2008 April; 70(17): 1549-54.

4. Lanoiselée HM, Nicolas G, Wallon D, Rovelet-Lecrux A, Lacour M, Rousseau S, et al. APP, PSEN1, and PSEN2 mutations in early-onset Alzheimer disease: A genetic screening study of familial and sporadic cases. PLoS Med. 2017. Mar;14(3).

5. Xia M, Chen S, Shi Y, Huang Y, Xu J, Zhao T, et al. Probable novel PSEN2 Pro123Leu mutation in a Chinese Han family of Alzheimer’s disease. Neurobiol Aging. 2015 Dec 1;36(12):3334.e13-3334.e18.

6. Müller U, Winter P, Bolender C, Nolte D. Previously Unrecognized Missense Mutation E126K of PSEN2 Segregates with Early Onset Alzheimer’s Disease in a Family. Journal of Alzheimer’s Disease. 2014;42(1):109–13.

7. Wang G, Zhang DF, Jiang HY, Fan Y, Ma L, Shen Z, et al. Mutation and association analyses of dementia-causal genes in Han Chinese patients with early-onset and familial Alzheimer’s disease. J Psychiatr Res. 2019. Jun;113:141–7.

8. Niu F, Yu S, Zhang Z, Yi X, Ye L, Tang W, et al. A novel mutation in the PSEN2 gene (N141Y) associated with early-onset autosomal dominant Alzheimer’s disease in a Chinese Han family. Neurobiol Aging. 2014;35(10):2420.e1-2420.e5.

9. Mao C, Li J, Dong L, Huang X, Lei D, Wang J, et al. Clinical Phenotype and Mutation Spectrum of Alzheimer’s Disease with Causative Genetic Mutation in a Chinese Cohort. Curr Alzheimer Res. 2021 Jun 9;18(3):265–72.

10. Shi Z, Wang Y, Liu S, Liu M, Liu S, Zhou Y, et al. Clinical and neuroimaging characterization of chinese dementia patients with psen1 and psen2 mutations. Dement Geriatr Cogn Disord. 2015. Feb 4;39:32–40.

11. Xu Y, Liu X, Shen J, Tian W, Fang R, Li B, et al. The whole exome sequencing clarifies the genotype- phenotype correlations in patients with early-onset dementia. Aging Dis. 2018;9(4):696–705.

12. Giau V Van, Pyun JM, Bagyinszky E, An SSA, Kim S. A pathogenic PSEN2 p.His169Asn mutation associated with early-onset Alzheimer’s disease. Clin Interv Aging. 2018;13:1321–9.

13. Guven G, Samanci B, Gulec C, Hanagasi H, Gurvit H, Gokalp EE, et al. A novel PSEN2 p.Ser175Phe variant in a family with Alzheimer’s disease. Neurological Sciences. 2021. Jun;42(6):2497–504.

14. Marín-Muñoz J, Noguera-Perea MF, Gómez-Tortosa E, López-Motos D, Antequera-Torres M, Martínez-Herrada B, et al. Novel Mutation (Gly212Val) in the PS2 Gene Associated with Early-Onset Familial Alzheimer’s Disease. Journal of Alzheimer’s Disease. 2016. Jun;53(1):73–8.

15. Lee JH, Kahn A, Cheng R, Reitz C, Vardarajan B, Lantigua R, et al. Disease-related mutations among caribbean hispanics with familial dementia. Mol Genet Genomic Med. 2014 Sep;2(5):430–7.

16. Sala Frigerio C, Lau P, Troakes C, Deramecourt V, Gele P, Van Loo P, et al. On the identification of low allele frequency mosaic mutations in the brains of Alzheimer’s disease patients. Alzheimers Dement. 2015. Nov;11(11):1265–76.

17. Hsu S, Gordon BA, Hornbeck R, Norton JB, Levitch D, Louden A, et al. Discovery and validation of autosomal dominant Alzheimer’s disease mutations. Alzheimers Res Ther. 2018. Jul;10(1). Available from: https://pubmed.ncbi.nlm.nih.gov/30021643/

18. Testi S, Fabrizi GM, Pompanin S, Cagnin A. Autosomal dominant alzheimer’s disease with early frontal lobe involvement associated with the Met239Ile mutation of presenilin 2 gene. Journal of Alzheimer’s Disease. 2012;31(1):7–11.

19. Tremolizzo L, Susani E, Mapelli C, Isella V, Bertola F, Ferrarese C, et al. First Report of PSEN2 Mutation Presenting as Posterior Cortical Atrophy. Alzheimer Disease and associated disorderds. 2015. Sept; 29 (3):249-51.

20. Llibre-Guerra JJ, Li Y, Allegri RF, Mendez PC, Surace EI, Llibre-Rodriguez JJ, et al. Dominantly inherited Alzheimer’s disease in Latin America: Genetic heterogeneity and clinical phenotypes. Alzheimer’s and Dementia. 2021. Apr;17(4):653–64.

21. Jiao B, Liu H, Guo L, Xiao X, Liao X, Zhou Y, et al. The role of genetics in neurodegenerative dementia: a large cohort study in South China. NPJ Genom Med. 2021. Dec;6(1).

22. Jia L, Fu Y, Shen L, Zhang H, Zhu M, Qiu Q, et al. PSEN1, PSEN2, and APP mutations in 404 Chinese pedigrees with familial Alzheimer’s disease. Alzheimer’s and Dementia. 2020 Jan;16(1):178–91.

23. Tedde A, Nacmias B, Ciantelli M, Forleo P, Cellini E, Bagnoli S, et al. Identification of new presenilin gene mutations in early-onset familial Alzheimer disease. Arch Neurol. 2003 Nov;60(11):1541–4.

24. Tomaino C, Bernardi L, Anfossi M, Costanzo A, Ferrise F, Gallo M, et al. Presenilin 2 Ser130Leu mutation in a case of late-onset “sporadic” Alzheimer’s disease. J Neurol. 2007 Mar;254(3):391–3.

25. Lohmann E, Guerreiro RJ, Erginel-Unaltuna N, Gurunlian N, Bilgic B, Gurvit H, et al. Identification of PSEN1 and PSEN2 gene mutations and variants in Turkish dementia patients. Neurobiol Aging. 2012;33(8).

26. Wojtas A, Heggeli KA, Finch NC, Baker M, DeJesus-Hernandez M, Younkin SG, et al. C9ORF72 repeat expansions and other FTD gene mutations in a clinical AD patient series from Mayo Clinic. Am J Neurodegener Dis. 2012.;1(1):107

27. Sassi C, Guerreiro R, Gibbs R, Ding J, Lupton MK, Troakes C, et al. Exome sequencing identifies 2 novel presenilin 1 mutations (p.L166V and p.S230R) in British early-onset Alzheimer’s disease. Neurobiol Aging. 2014;35(10):2422.e13-2422.e16.

28. Schulte EC, Fukumori A, Mollenhauer B, Hor H, Arzberger T, Perneczky R, et al. Rare variants in β-Amyloid precursor protein (APP) and Parkinson’s disease. Eur J Hum Genet. 2015.Oct; 23(10):1328–33.

29. Nicolas G, Wallon D, Charbonnier C, Quenez O, Rousseau S, Richard AC, et al. Screening of dementia genes by whole-exome sequencing in early-onset Alzheimer disease: input and lessons. Eur J Hum Genet. 2016. May;24(5):710–6.

30. Wallon D, Rousseau S, Rovelet-Lecrux A, Quillard-Muraine M, Guyant-Maréchal L, Martinaud O, et al. The French series of autosomal dominant early onset Alzheimer’s disease cases: mutation spectrum and cerebrospinal fluid biomarkers. J Alzheimers Dis. 2012;30(4):847–56.

31. Levy-Lahad E, Wasco W, Poorkaj P, Romano DM, Oshima J, Pettingell WH, et al. Candidate gene for the chromosome 1 familial Alzheimer’s disease locus. Science. 1995;269(5226):973–7.

32. Blauwendraat C, Wilke C, Jansen IE, Schulte C, Simón-Sánchez J, Metzger FG, et al. Pilot whole-exome sequencing of a German early-onset Alzheimer’s disease cohort reveals a substantial frequency of PSEN2 variants. Neurobiol Aging. 2016 Jan;37:208.e11-208.e17.

33. Marcon G, Giaccone G, Cupidi C, Balestrieri M, Beltrami CA, Finato N, et al. Neuropathological and clinical phenotype of an Italian Alzheimer family with M239V mutation of presenilin 2 gene. J Neuropathol Exp Neurol. 2004;63(3):199–209.

34. Li C, Xiao X, Wang J, Shen L, Jiao B. Early - onset familial Alzheimer ’ s disease in a family with mutation of presenilin 2 gene. Zhong Nan Da Xue Xue Bao Yi Xue Ban. 2021. Feb;46(2):189–94.

35. Jiao B, Liu H, Guo L, Xiao X, Liao X, Zhou Y, et al. The role of genetics in neurodegenerative dementia: a large cohort study in South China. NPJ Genom Med. 2021 Dec 1;6(1).

36. Dobricic V, Stefanova E, Jankovic M, Gurunlian N, Novakovic I, Hardy J, et al. Genetic testing in familial and young-onset Alzheimer’s disease: mutation spectrum in a Serbian cohort. Neurobiol Aging. 2012 Jul 1;33(7):1481.e7-1481.e12.

37. Sorbi S, Tedde A, Nacmias B, Ciantelli M, Caffarra P, Ghidoni E, Bracco L, Piccini C. Novel presenilin 1 and presenilin 2 mutations in early-onset Alzheimer's disease families. Neurobiol Aging. 2002 Jul-Aug;23(1S):312.

38. Llibre-Guerra JJ, Li Y, Allegri RF, Mendez PC, Surace EI, Llibre-Rodriguez JJ, Sosa AL, Aláez-Verson C, Longoria EM, Tellez A, Carrillo-Sánchez K, Flores-Lagunes LL, Sánchez V, Takada LT, Nitrini R, Ferreira-Frota NA, Benevides-Lima J, Lopera F, Ramírez L, Jiménez-Velázquez I, Schenk C, Acosta D, Behrens MI, Doering M, Ziegemeier E, Morris JC, McDade E, Bateman RJ. Dominantly inherited Alzheimer's disease in Latin America: Genetic heterogeneity and clinical phenotypes. Alzheimers Dement. 2021 Apr;17(4):653-664.

39. Muchnik C, Olivar N, Dalmasso MC, Azurmendi PJ, Liberczuk C, Morelli L, et al. Identification of PSEN2 mutation p.N141I in Argentine pedigrees with early-onset familial Alzheimer’s disease. Neurobiol Aging. 2015 Oct1 ;36(10):2674-2677.

40. Perrone F, Bjerke M, Hens E, Sieben A, Timmers M, De Roeck A, Vandenberghe R, Sleegers K, Martin JJ, De Deyn PP, Engelborghs S, van der Zee J, Van Broeckhoven C, Cacace R, BELNEU Consortium. Amyloid-β1-43 cerebrospinal fluid levels and the interpretation of APP, PSEN1 and PSEN2 mutations. Alzheimers Res Ther. 2020 Sep 11;12(1):108.

41. Piscopo P, Talarico G, Crestini A, Gasparini M, Malvezzi-Campeggi L, Piacentini E, Lenzi GL, Bruno G, Confaloni A. A novel mutation in the predicted TMIII domain of the PSEN2 gene in an Italian pedigree with atypical Alzheimer's disease. J Alzheimers Dis. 2010;20(1):43-7.

42. Sleegers K, Roks G, Theuns J, Aulchenko YS, Rademakers R, Cruts M, van Gool WA, Van Broeckhoven C, Heutink P, Oostra BA, van Swieten JC, van Duijn CM. Familial clustering and genetic risk for dementia in a genetically isolated Dutch population. Brain. 2004 Jul;127(Pt 7):1641-9.

43. Coppola C, Saracino D, Oliva M, Cipriano L, Puoti G, Pappatà S, Di Fede G, Catania M, Ricci M, Cimini S, Giaccone G, Bonavita S, Rossi G. Singular cases of Alzheimer's disease disclose new and old genetic "acquaintances". Neurol Sci. 2020 Oct 2.

44. Youn YC, Bagyinszky E, Kim H, Choi BO, An SS, Kim S. Probable novel PSEN2 Val214Leu mutation in Alzheimer's disease supported by structural prediction. BMC Neurol. 2014 May 15;14:105.

45. An SS, Park SA, Bagyinszky E, Bae SO, Kim YJ, Im JY, Park KW, Park KH, Kim EJ, Jeong JH, Kim JH, Han HJ, Choi SH, Kim S. A genetic screen of the mutations in the Korean patients with early-onset Alzheimer's disease. Clin Interv Aging. 2016;11:1817-1822.

46. Yagi R, Miyamoto R, Morino H, Izumi Y, Kuramochi M, Kurashige T, Maruyama H, Mizuno N, Kurihara H, Kawakami H. Detecting gene mutations in Japanese Alzheimer's patients by semiconductor sequencing. Neurobiol Aging. 2014 Jul;35(7):1780.e1-5.

**References Table S2 (APP)**

1. Obici L, Demarchi A, De Rosa G, Bellotti V, Marciano S, Donadei S, et al. A novel AbetaPP mutation exclusively associated with cerebral amyloid angiopathy. Ann Neurol. 2005 Oct;58(4):639–44.

2. Kozberg MG, Van Veluw SJ, Frosch MP, Greenberg SM. Hereditary cerebral amyloid angiopathy, Piedmont-type mutation. Neurol Genet. 2020;6(2). Available from:

3. Schulte EC, Fukumori A, Mollenhauer B, Hor H, Arzberger T, Perneczky R, et al. Rare variants in β-Amyloid precursor protein (APP) and Parkinson’s disease. Eur J Hum Genet. 2015. Oct;23(10):1328–33.

4. Carter DA, Desmarais E, Bellis M, Campion D, Clerget-Darpoux F, Brice A, et al. More missense in amyloid gene. Nat Genet [Internet]. 1992.;2(4):255–6.

5. Rossi G, Giaccone G, Maletta R, Morbin M, Capobianco R, Mangieri M, et al. A family with Alzheimer disease and strokes associated with A713T mutation of the APP gene. Neurology. 2004 Sep;63(5):910–2.

6. Armstrong J, Boada M, Rey MJ, Vidal N, Ferrer I. Familial Alzheimer disease associated with A713T mutation in APP. Neurosci Lett. 2004 Nov;370(2–3):241–3.

7. Conidi ME, Bernardi L, Puccio G, Smirne N, Muraca MG, Curcio SAM, et al. Homozygous carriers of APP A713T mutation in an autosomal dominant Alzheimer disease family. Neurology. 2015 Jun;84(22):2266–73.

8. Barber IS, García-Cárdenas JM, Sakdapanichkul C, Deacon C, Zapata Erazo G, Guerreiro R, et al. Screening exons 16 and 17 of the amyloid precursor protein gene in sporadic early-onset Alzheimer’s disease. Neurobiol Aging. 2016 Mar 1;39:220.e1-220.e7.

9. Lombardi G, Berti V, Tedde A, Bagnoli S, Piaceri I, Polito C, et al. Low Florbetapir PET Uptake and Normal Aβ1-42 Cerebrospinal Fluid in an APP Ala713Thr Mutation Carrier. J Alzheimers Dis. 2017;57(3):697–703.

10. Jones CT, Morris S, Yates CM, Moffoot A, Sharpe C, Brock DJH, et al. Mutation in codon 713 of the beta amyloid precursor protein gene presenting with schizophrenia. Nat Genet. 1992;1(4):306–9.

11. Zekanowski C, Styczyńska M, Pepłońska B, Gabryelewicz T, Religa D, Ilkowski J, et al. Mutations in presenilin 1, presenilin 2 and amyloid precursor protein genes in patients with early-onset Alzheimer’s disease in Poland. Exp Neurol. 2003;184(2):991–6.

12. Lindquist SG, Nielsen JE, Stokholm J, Schwartz M, Batbayli M, Ballegaard M, et al. Atypical early-onset Alzheimer’s disease caused by the Iranian APP mutation. J Neurol Sci. 2008 May;268(1–2):124–30.

13. Pasalar P, Najmabadi H, Noorian AR, Moghimi B, Jannati A, Soltanzadeh A, et al. An Iranian family with Alzheimer’s disease caused by a novel APP mutation (Thr714Ala). Neurology. 2002 May; 58(10):1574–5.

14. Kumar-Singh S, De Jonghe C, Cruts M, Kleinert R, Wang R, Mercken M, et al. Nonfibrillar diffuse amyloid deposition due to a gamma(42)-secretase site mutation points to an essential role for N-truncated A beta(42) in Alzheimer’s disease. Hum Mol Genet. 2000 Nov;9(18):2589–98.

15. Edwards-Lee T, Ringman JM, Chung J, Werner J, Morgan A, St. George Hyslop P, et al. An African American family with early-onset Alzheimer disease and an APP (T714I) mutation. Neurology. 2005 Jan;64(2):377–9.

16. Cruts M, Dermaut B, Rademakers R, Van Den Broeck M, Stögbauer F, Van Broeckhoven C. Novel APP mutation V715A associated with presenile Alzheimer’s disease in a German family. J Neurol. 2003 Nov;250(11):1374–5.

17. Wallon D, Rousseau S, Rovelet-Lecrux A, Quillard-Muraine M, Guyant-Maréchal L, Martinaud O, et al. The French series of autosomal dominant early onset Alzheimer’s disease cases: mutation spectrum and cerebrospinal fluid biomarkers. J Alzheimers Dis. 2012;30(4):847–56.

18. Ancolio K, Dumanchin C, Barelli H, Warter JM, Brice A, Campion D, et al. Unusual phenotypic alteration of beta amyloid precursor protein (betaAPP) maturation by a new Val-715 --> Met betaAPP-770 mutation responsible for probable early-onset Alzheimer’s disease. Proc Natl Acad Sci U S A. 1999. Mar;96(7):4119–24.

19. Park HK, Duk LN, Lee JH, Kim JW, Ki CS. Identification of PSEN1 and APP gene mutations in Korean patients with early-onset Alzheimer’s disease. J Korean Med Sci [Internet]. 2008. Apr;23(2):213–7.

20. Guerreiro RJ, Baquero M, Blesa R, Boada M, Brás JM, Bullido MJ, et al. Genetic screening of Alzheimer’s disease genes in Iberian and African samples yields novel mutations in presenilins and APP. Neurobiol Aging. 2010 May;31(5):725–31.

21. Sieczkowski E, Milenkovic I, Venkataramani V, Giera R, Ströbel T, Höftberger R, et al. I716F AβPP mutation associates with the deposition of oligomeric pyroglutamate amyloid-β and α-synucleinopathy with Lewy bodies. J Alzheimers Dis. 2015;44(1):103–14.

22. Blauwendraat C, Wilke C, Jansen IE, Schulte C, Simón-Sánchez J, Metzger FG, et al. Pilot whole-exome sequencing of a German early-onset Alzheimer’s disease cohort reveals a substantial frequency of PSEN2 variants. Neurobiol Aging [Internet]. 2016.Jan;37:208.e11-208.e17.

23. Eckman CB, Mehta ND, Crook R, Perez-tur J, Prihar G, Pfeiffer E, et al. A new pathogenic mutation in the APP gene (I716V) increases the relative proportion of A beta 42(43). Hum Mol Genet. 1997 Nov;6(12):2087–9.

24. Finckh U, Kuschel C, Anagnostouli M, Patsouris E, Pantes G V., Gatzonis S, et al. Novel mutations and repeated findings of mutations in familial Alzheimer disease. Neurogenetics. 2005 May;6(2):85–9.

25. Murrell J, Farlow M, Ghetti B, Benson MD. A mutation in the amyloid precursor protein associated with hereditary Alzheimer’s disease. Science. 1991;254(5028):97–9.

26. Zádori D, Füvesi J, Timár E, Horváth E, Bencsik R, Szépfalusi N, et al. The Report of p.Val717Phe Mutation in the APP Gene in a Hungarian Family With Alzheimer Disease: A Phenomenological Study. Alzheimer Dis Assoc Disord [Internet]. 2017;31(4):343–5.

27. Abe M, Sonobe N, Fukuhara R, Mori Y, Ochi S, Matsumoto T, et al. Phenotypical difference of Amyloid Precursor Protein (APP) V717L mutation in Japanese family. BMC Neurol. 2012 Jun 15;12(1):1–5.

28. Chartier-Harlin MC, Crawford F, Houlden H, Warren A, Hughes D, Fidani L, et al. Early-onset Alzheimer’s disease caused by mutations at codon 717 of the beta-amyloid precursor protein gene. Nature. 1991;353(6347):844–6.

29. Küçükali F, Neumann A, Van Dongen J, De Pooter T, Joris G, De Rijk P, et al. Whole-exome rare-variant analysis of Alzheimer’s disease and related biomarker traits. Alzheimers Dement. 2023 Jun;19(6):2317–31.

30. Mullan M, Tsuji S, Miki T, Katsuya T, Naruse S, Kaneko K, et al. Clinical comparison of Alzheimer’s disease in pedigrees with the codon 717 Val→Ile mutation in the amyloid precursor protein gene. Neurobiol Aging. 1993 Sep 1;14(5):407–19.

31. Zhang G, Xie Y, Wang W, Feng X, Jia J. Clinical characterization of an APP mutation (V717I) in five Han Chinese families with early-onset Alzheimer’s disease. J Neurol Sci. 2017 Jan 15;372:379–86.

32. Murrell JR, Hake AM, Quaid KA, Farlow MR, Ghetti B. Early-Onset Alzheimer Disease Caused by a New Mutation (V717L) in the Amyloid Precursor Protein Gene. Arch Neurol. 2000 Jun 1;57(6):885–7.

33. Godbolt AK, Beck JA, Collinge JC, Cipolotti L, Fox NC, Rossor MN. A second family with familial AD and the V717L APP mutation has a later age at onset. Neurology. 2006 Feb;66(4):611–2.

34. Sassi C, Guerreiro R, Gibbs R, Ding J, Lupton MK, Troakes C, et al. Exome sequencing identifies 2 novel presenilin 1 mutations (p.L166V and p.S230R) in British early-onset Alzheimer’s disease. Neurobiol Aging. 2014 Oct 1;35(10):2422.e13-2422.e16.

35. Hooli B V., Mohapatra G, Mattheisen M, Parrado AR, Roehr JT, Shen Y, et al. Role of common and rare APP DNA sequence variants in Alzheimer disease. Neurology. 2012 Apr 17;78(16):1250–7.

36. Ghetti B, Hake AM, Murrell JR, Epperson F, Farlow MR, Vidal R, et al. P3‐221: Familial Alzheimer’s disease associated with the V717L amyloid precursor protein gene mutation: Neuropathological characterization. Alzheimer’s & Dementia. 2008 Jul;4(4S_Part_18).

37. Scahill RI, Ridgway GR, Bartlett JW, Barnes J, Ryan NS, Mead S, et al. Genetic Influences on Atrophy Patterns in Familial Alzheimer’s Disease: A Comparison of APP and PSEN1 Mutations. Journal of Alzheimer’s Disease. 2013 Jan 1;35(1):199–212.

38. Ghidoni R, Albertini V, Squitti R, Paterlini A, Bruno A, Bernardini S, et al. Novel T719P AβPP Mutation Unbalances the Relative Proportion of Amyloid-β Peptides. Journal of Alzheimer’s Disease. 2009 Jan 1;18(2):295–303.

39. Wang Q, Jia J, Qin W, Wu L, Li D, Wang Q, et al. A Novel AβPP M722K Mutation Affects Amyloid-β Secretion and Tau Phosphorylation and May Cause Early-Onset Familial Alzheimer’s Disease in Chinese Individuals. Journal of Alzheimer’s Disease. 2015 Jan 1;47(1):157–65.

40. Hsu S, Gordon BA, Hornbeck R, Norton JB, Levitch D, Louden A, et al. Discovery and validation of autosomal dominant Alzheimer’s disease mutations. Alzheimers Res Ther. 2018 Jul 18 [cited 2024 Aug 14];10(1).

41. Dobricic V, Stefanova E, Jankovic M, Gurunlian N, Novakovic I, Hardy J, et al. Genetic testing in familial and young-onset Alzheimer’s disease: mutation spectrum in a Serbian cohort. Neurobiol Aging. 2012 Jul 1;33(7):1481.e7-1481.e12.

42. John B. J. Kwok, Qiao-Xin Li, Marianne Hallupp, Scott Whyte, David Ames, Konrad Beyreuther, et al. Annals of Neurology . 2000. p. 2–8 Novel Leu723Pro amyloid precursor protein mutation increases amyloid beta42(43) peptide levels and induces apoptosis - PubMed.

43. Theuns J, Marjaux E, Vandenbulcke M, Van Laere K, Kumar-Singh S, Bormans G, et al. Alzheimer dementia caused by a novel mutation located in the APP C-terminal intracytosolic fragment. Hum Mutat. 2006 Sep 1;27(9):888–96.

44. Terreni L, Fogliarino S, Franceschi M, Forloni G. Novel pathogenic mutation in an Italian patient with familial Alzheimer's disease detected in APP gene. Neurobiol Aging. 2002 Jul-Aug;23(1S):319.

45. Wang Q, Jia J, Qin W, Wu L, Li D, Wang Q, Li H. A Novel AβPP M722K Mutation Affects Amyloid-β Secretion and Tau Phosphorylation and May Cause Early-Onset Familial Alzheimer's Disease in Chinese Individuals. J Alzheimers Dis. 2015;47(1):157-65.

**References Table S3 (PSEN1)**

1. Petit D, Fernández SG, Zoltowska KM, Enzlein T, Ryan NS, O’Connor A, et al. Aβ profiles generated by Alzheimer’s disease causing PSEN1 variants determine the pathogenicity of the mutation and predict age at disease onset. Molecular Psychiatry 2022 27:6. 2022 Apr 1;27(6):2821–32.

2. Ringman JM, Dorrani N, Fernández SG, Signer R, Martinez-Agosto J, Lee H, et al. Characterization of spastic paraplegia in a family with a novel PSEN1 mutation. Brain Commun. 2023 Mar 2;5(2).

3. Robbie L, Fernández SG, Montoya L, Sagare A, Barrera L, Sheikh-Bahaei N, et al. Age of onset predicted by Aβ profiling in a novel PSEN1 (I180F) mutation. Neurosci Lett. 2024 Jan 18;820.

4. Dermaut B, Kumar-Singh S, Engelborghs S, Theuns J, Rademakers R, Saerens J, et al. A novel presenilin 1 mutation associated with Pick’s disease but not beta-amyloid plaques. Ann Neurol. 2004 May;55(5):617–26.

5. Eryilmaz IE, Bakar M, Egeli U, Cecener G, Yurdacan B, Colak DK, et al. Evaluation of the Clinical Features Accompanied by the Gene Mutations: The 2 Novel PSEN1 Variants in a Turkish Early-onset Alzheimer Disease Cohort. Alzheimer Dis Assoc Disord. 2021 Jul 1;35(3):214–22.

6. Koriath C, Kenny J, Adamson G, Druyeh R, Taylor W, Beck J, et al. Predictors for a dementia gene mutation based on gene-panel next-generation sequencing of a large dementia referral series. Mol Psychiatry. 2020 Dec 1;25(12):3399–412.

7. Matsubara-Tsutsui M, Yasuda M, Yamagata H, Nomura T, Taguchi K, Kohara K, et al. Molecular evidence of presenilin 1 mutation in familial early onset dementia. Am J Med Genet. 2002 Apr 8;114(3):292–8.

8. Kim YE, Cho H, Kim HJ, Na DL, Seo SW, Ki CS. PSEN1 variants in Korean patients with clinically suspicious early-onset familial Alzheimer’s disease. Sci Rep. 2020 Dec 1;10(1).

9. Chelban V, Breza M, Szaruga M, Vandrovcova J, Murphy D, Lee CJ, et al. Spastic paraplegia preceding PSEN1-related familial Alzheimer’s disease. Alzheimers Dement (Amst). 2021;13(1).

10. Dumanchin C, Tournier I, Martin C, Didic M, Belliard S, Carlander B, et al. Biological effects of four PSEN1 gene mutations causing Alzheimer disease with spastic paraparesis and cotton wool plaques. Hum Mutat. 2006;27(10):1063.

11. Hattori S, Sakuma K, Wakutani Y, Wada K, Shimoda M, Urakami K, et al. A novel presenilin 1 mutation (Y154N) in a patient with early onset Alzheimer’s disease with spastic paraparesis. Neurosci Lett. 2004 Sep 30;368(3):319–22.

12. Raux G, Gantier R, Thomas-Anterion C, Boulliat J, Verpillat P, Hannequin D, et al. Dementia with prominent frontotemporal features associated with L113P presenilin 1 mutation. Neurology. 2000 Nov 28;55(10):1577–8.

13. Heilig EA, Xia W, Shen J, Kelleher RJ. A presenilin-1 mutation identified in familial Alzheimer disease with cotton wool plaques causes a nearly complete loss of gamma-secretase activity. J Biol Chem. 2010 Jul 16;285(29):22350–9.

14. Koriath C, Kenny J, Adamson G, Druyeh R, Taylor W, Beck J, et al. Predictors for a dementia gene mutation based on gene-panel next-generation sequencing of a large dementia referral series. Mol Psychiatry. 2020 Dec 1;25(12):3399–412.

15. Campion D, Flaman JM, Brice A, Hannequin D, Dubois B, Martin C, et al. Mutations of the presenilin I gene in families with early-onset Alzheimer’s disease. Hum Mol Genet. 1995 Dec;4(12):2373–7.

16. Sherrington R, Rogaev EI, Liang Y, Rogaeva EA, Levesque G, Ikeda M, et al. Cloning of a gene bearing missense mutations in early-onset familial Alzheimer’s disease. Nature. 1995;375(6534):754–60.

17. Godbolt AK, Beck JA, Collinge J, Garrard P, Warren JD, Fox NC, et al. A presenilin 1 R278I mutation presenting with language impairment. Neurology. 2004 Nov 9;63(9):1702–4.

18. Islam S, Sun Y, Gao Y, Nakamura T, Noorani AA, Li T, et al. Presenilin Is Essential for ApoE Secretion, a Novel Role of Presenilin Involved in Alzheimer’s Disease Pathogenesis. J Neurosci. 2022 Feb 23;42(8):1574–86.

19. Vázquez-Costa JF, Payá-Montes M, Martínez-Molina M, Jaijo T, Szymanski J, Mazón M, et al. Presenilin-1 Mutations Are a Cause of Primary Lateral Sclerosis-Like Syndrome. Front Mol Neurosci. 2021 Aug 30;14.

20. Liu CY, Ohki Y, Tomita T, Osawa S, Reed BR, Jagust W, et al. Two Novel Mutations in the First Transmembrane Domain of Presenilin1 Cause Young-Onset Alzheimer’s Disease. J Alzheimers Dis. 2017;58(4):1035–41.

21. Goudsmit J, White BJ, Weitkamp LR, Keats BJB, Morrow CH, Gajdusek DC. Familial Alzheimer’s disease in two kindreds of the same geographic and ethnic origin. A clinical and genetic study. J Neurol Sci. 1981;49(1):79–89.
