# Supplementary figures and images for: Spectrum of γ-Secretase dysfunction as a unifying predictor of ADAD age at onset across PSEN1, PSEN2 and APP causal genes
Source: Mol Neurodegener. 2025 Apr 26;20:48. doi: 10.1186/s13024-025-00832-1 (PMC12032737; doi:10.1186/s13024-025-00832-1)

## Slide 1
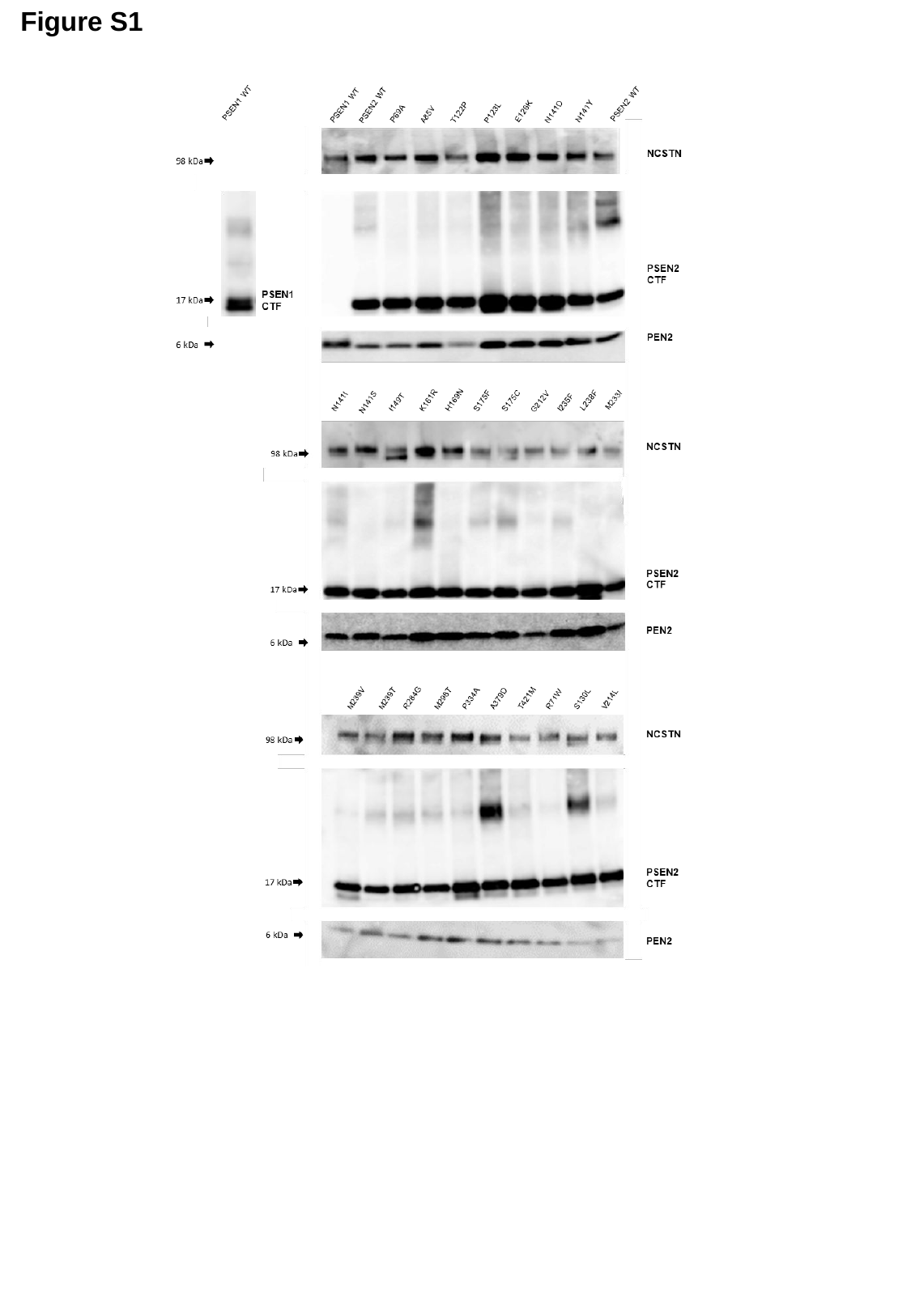

Figure S1

## Slide 2
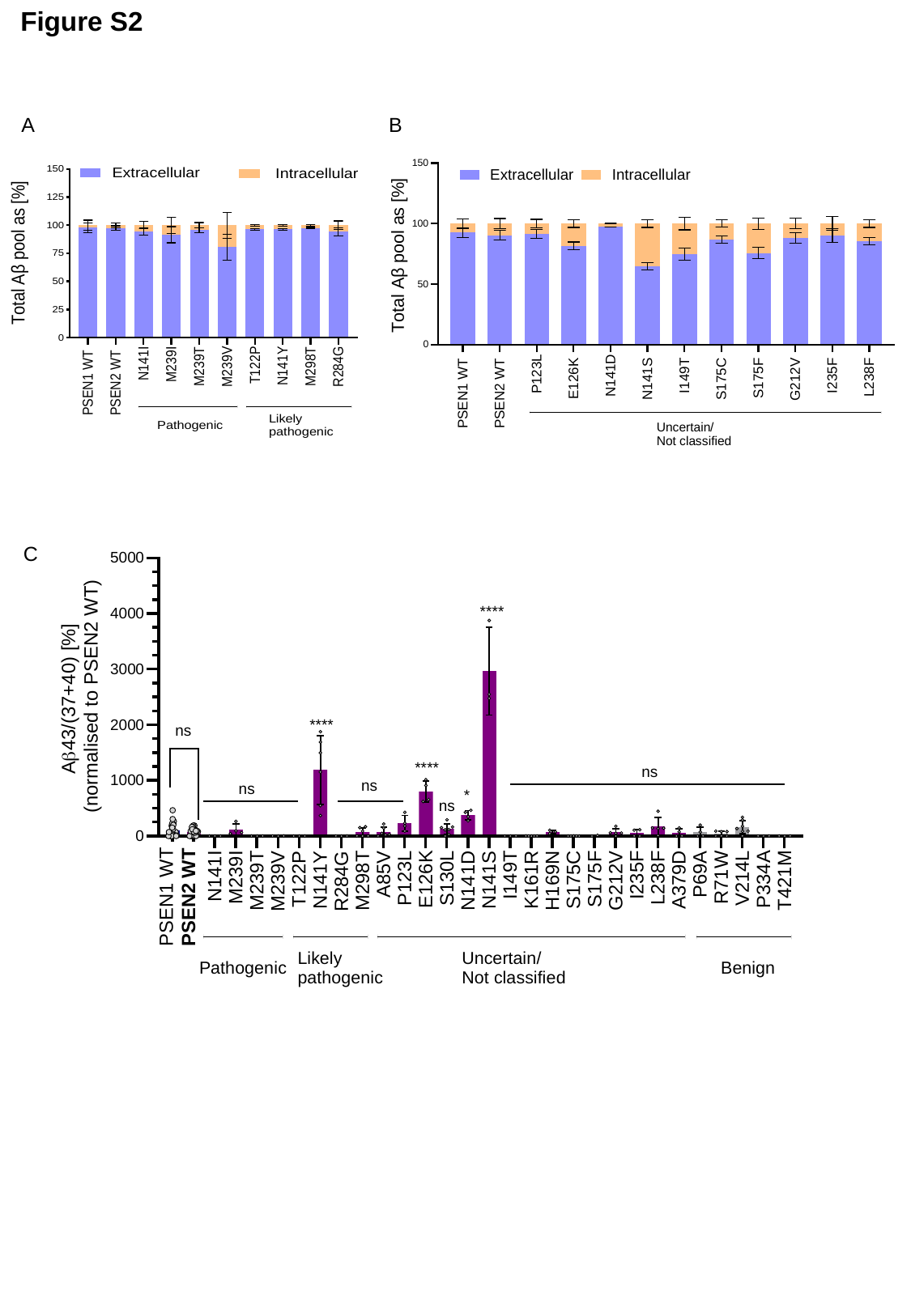

Figure S2
A
B
C

## Slide 3
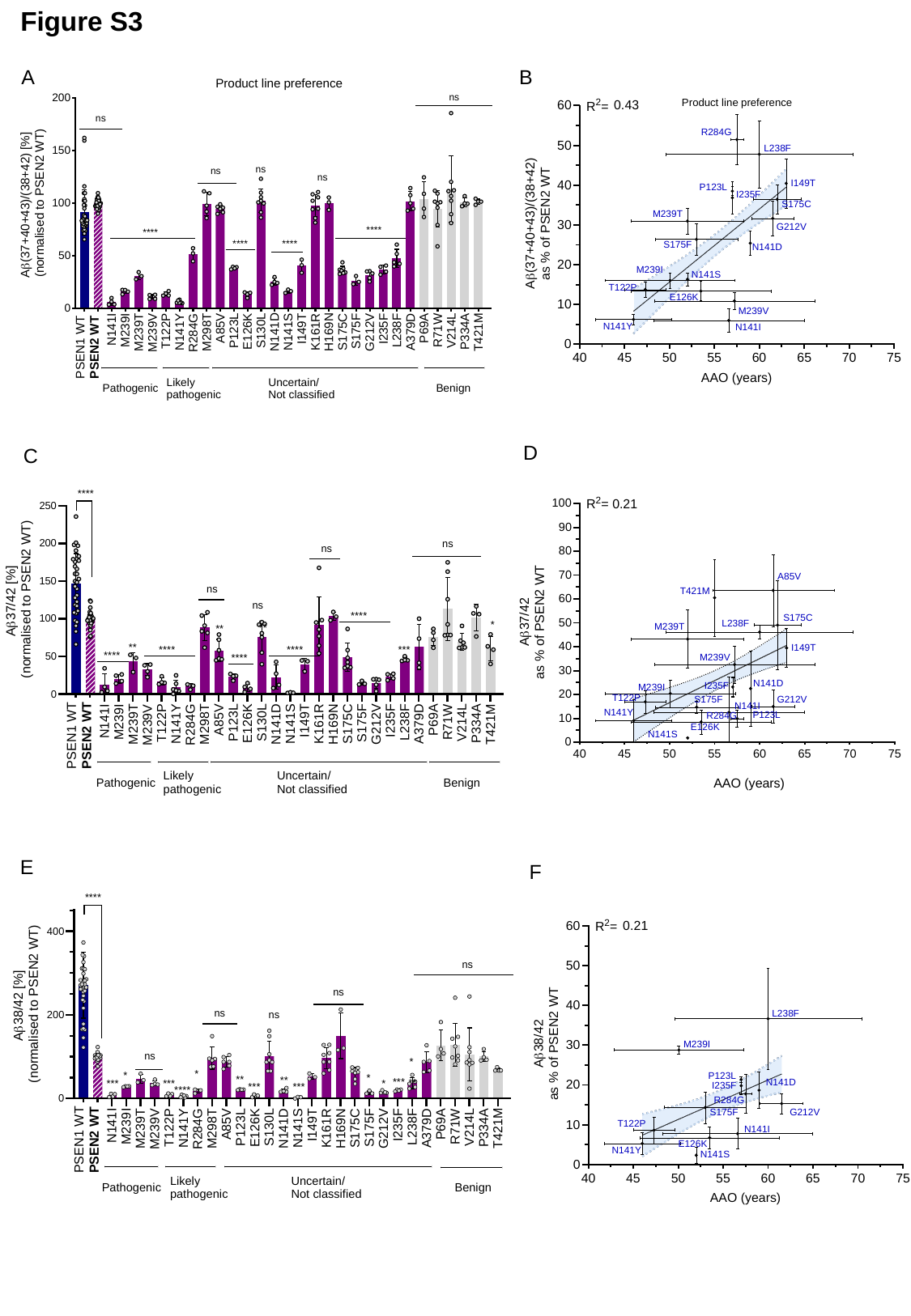

Figure S3
A
B
D
C
E
F

## Slide 4
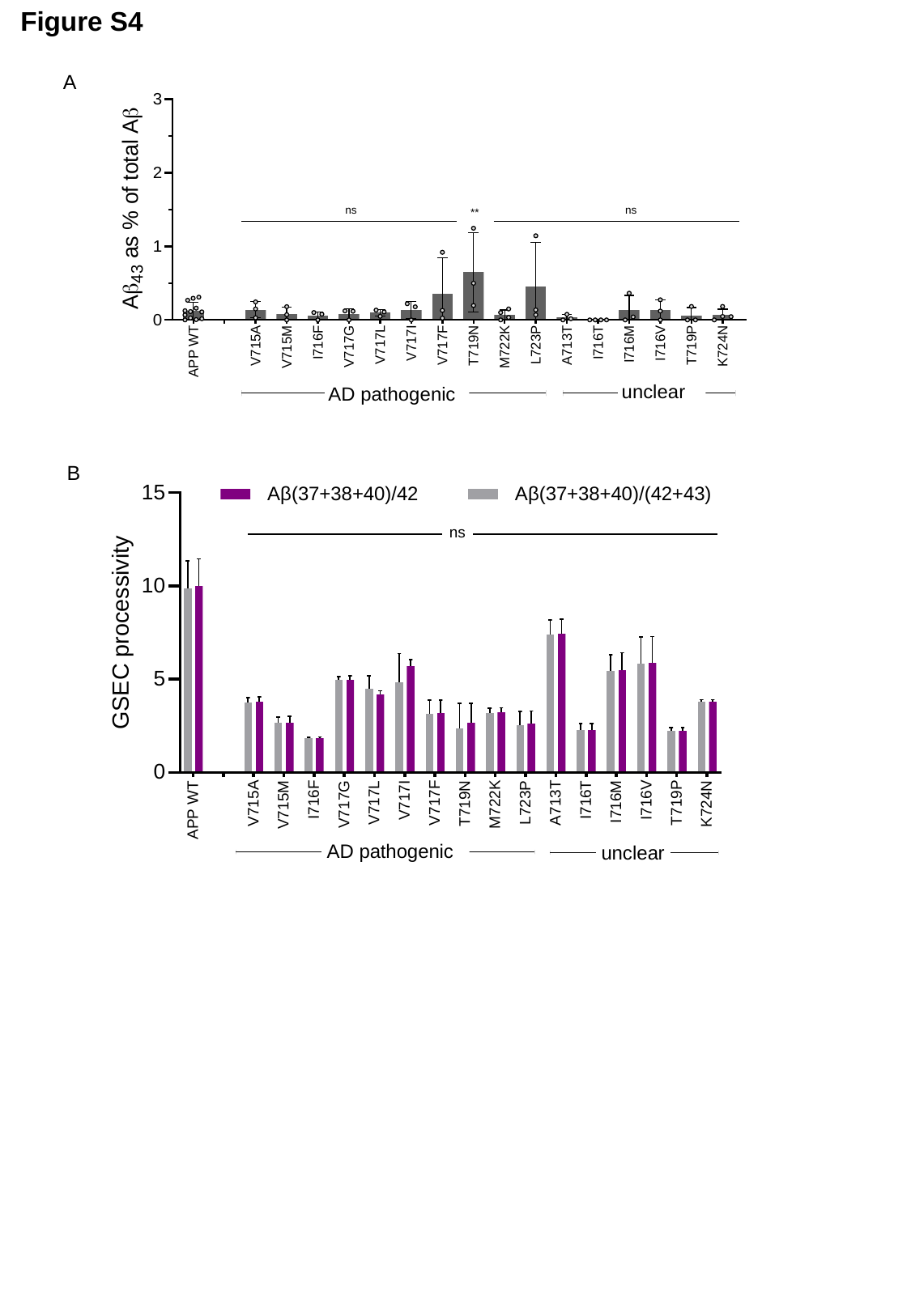

Figure S4
A
B

## Slide 5
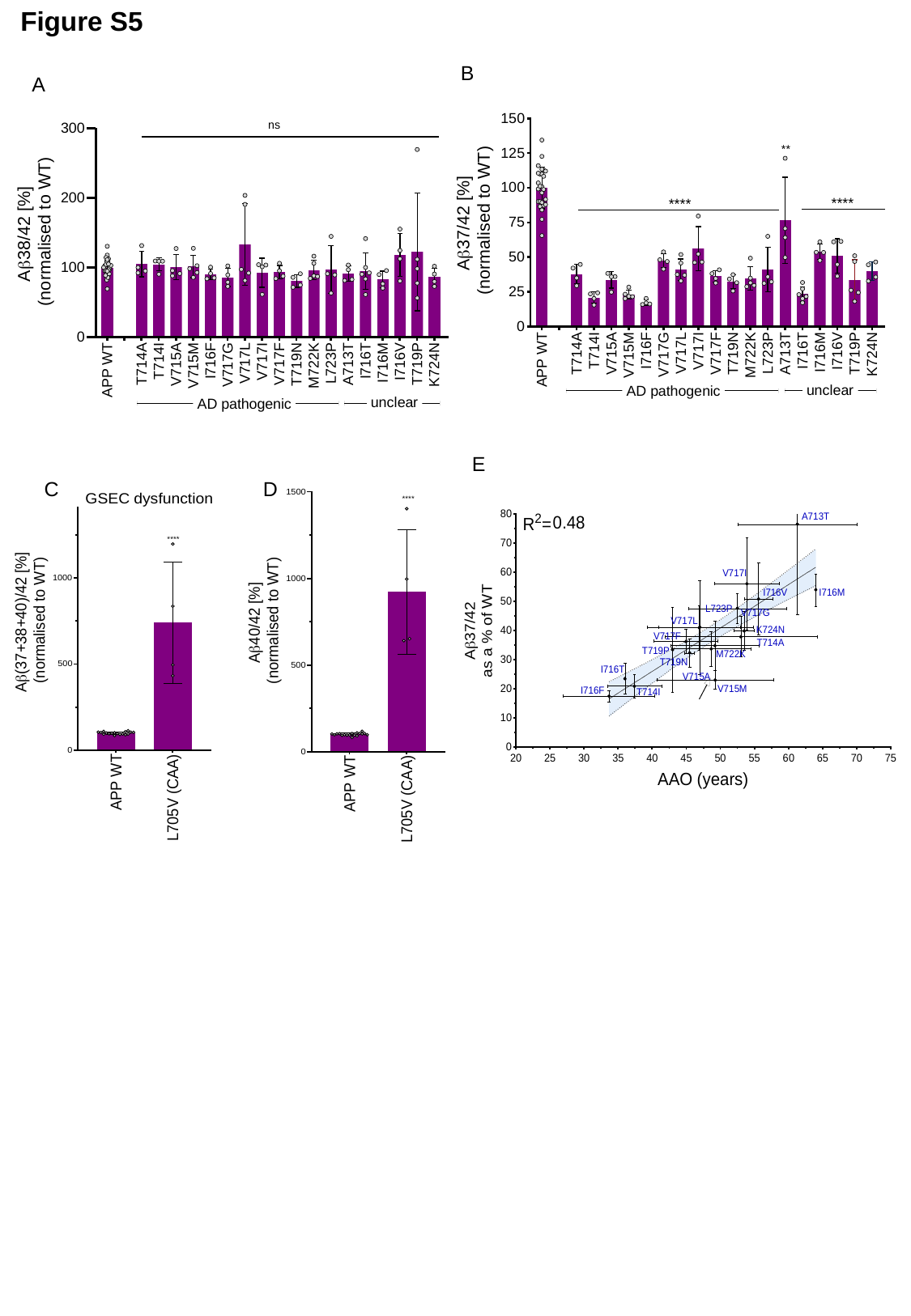

Figure S5
B
A
E
C
D

Supplement: Supplementary file 2 — Supplementary Material 2. [file 13024_2025_832_MOESM2_ESM.pptx]
